# Genetic and Physiological Dissection of Photosynthesis in Barley Exposed to Drought Stress

**DOI:** 10.3390/ijms20246341

**Published:** 2019-12-16

**Authors:** Agata Daszkowska-Golec, Anna Collin, Krzysztof Sitko, Agnieszka Janiak, Hazem M. Kalaji, Iwona Szarejko

**Affiliations:** 1 Institute of Biology, Biotechnology and Environmental Protection, Faculty of Natural Sciences, University of Silesia in Katowice, Jagiellońska 28, 40-032 Katowice, Poland; 2 Department of Plant Physiology, Institute of Biology, Warsaw University of Life Sciences (WULS-SGGW), Nowoursynowska 159, 02-776 Warszawa, Poland

**Keywords:** abiotic stress, barley, drought stress, JIP-test, photosynthesis, transcriptome

## Abstract

Balanced photosynthesis under drought is essential for better survival and for agricultural benefits in terms of biomass and yield. Given the current attempts to improve the photosynthetic efficiency for greater crop yield, the explanation of the genetic basis of that process, together with the phenotypic analysis, is significant in terms of both basic studies and potential agricultural application. Therefore, the main objective of this study was to uncover the molecular basis of the photosynthesis process under drought stress in barley. To address that goal, we conducted transcriptomic examination together with detailed photosynthesis analysis using the JIP-test. Using this approach, we indicated that photosynthesis is a process that is very early affected in barley seedlings treated with severe drought stress. Rather than focusing on individual genes, our strategy was pointed to the identification of groups of genes with similar expression patterns. As such, we identified and annotated almost 150 barley genes as crucial core-components of photosystems, electron transport components, and Calvin cycle enzymes. Moreover, we designated 17 possible regulatory interactions between photosynthesis-related genes and transcription factors in barley. Summarizing, our results provide a list of candidate genes for future genetic research and improvement of barley drought tolerance by targeting photosynthesis.

## 1. Introduction

In the present climate change scenarios, water deficit is the main environmental stress that negatively influences crop yield. It is recognized as a global problem that threatens the world’s food security, taking into account both shrinking agricultural areas and constantly reduced the production of major crops. Currently, it is of great significance to elaborate the promising strategies to obtain crops with the ability to adapt and to tolerate water deficit and at the same time, achieve appropriate yield under these critical conditions.

Plants’ response to water stress involves a very complex regulatory network consisting of many pathways that interact with each other at many different points leading to adaptation to harsh environmental conditions [1,2]. In response to water deficit, plants close partially or completely their stomata to limit transpiration [3]. Although that strategy evolved as the most rapid water-saving mechanism, it comes at the cost of restriction of CO_2_ assimilation ability [4], which is extremely important for organic matter production in the photosynthesis process [5,6,7]. Consequently, a slower or inhibited CO_2_ assimilation diminishes the photosynthetic rate. The secondary oxidative stress caused by drought also influences the photosystem II (PSII) activity negatively. This is caused mainly by the production of Reactive Oxygen Species (ROS) that inhibit PSII repair [8]. Photosynthesis consists of two phases: light reactions occurring in thylakoids and the carbon reduction reactions that take place in chloroplast stroma [9]. Thylakoid membranes include four main protein complexes: photosystem I (PSI), photosystem II (PSII), cytochrome b6f complex (Cytb6f), and adenosine triphosphate (ATP) synthase. Reactions of the dark part of photosynthesis involve the key enzyme—ribulose-1,5-bisphosphate carboxylase (Rubisco) [10].

PSI and PSII are associated with their light-absorbing antenna systems, light-harvesting chlorophyll-binding I (LHCI), and light-harvesting chlorophyll-binding II (LHCII), respectively. LHCI contains mainly chlorophyll *a* while LHCII is enriched with chlorophyll *b* [11]. Photonic energy is absorbed by LHCs and further transferred to PS reaction centers. Light-driven charge separation in the reaction centers of PSII and PSI initiates an electron transport coupled with proton transport that builds up the proton motive force [12,13]. In PSII, the splitting of water at the oxygen-evolving complex (OEC) produces H^+^ ions in the lumen of the thylakoid that leads to acidification of the lumen matrix, while electrons (e-) are transferred to PSI via Cytb6f complex and plastocyanin (PC) pool. Electrons that are removed from the water are further transferred to the single-electron carrier ferredoxin (Fd). Next, ferredoxin NADP^+^ reductase (FNR) transfers an electron from each of the two Fd molecules to the single-molecule of nicotinamide adenine dinucleotide phosphate H (NADPH). H+ ions are transported back to the stroma by the ATP synthase, which converts the energy of the H+ gradient into chemical energy in the form of ATP [8,12,13].

Under normal and stressed conditions, PSII efficiency can be evaluated by the use of a non-destructive method such as fast chlorophyll transient kinetics. The chlorophyll *a* fluorescence (ChlF) analysis that allows for tracking changes in the photosynthetic apparatus and thus these measurements are documented as a reliable tool in assessing the sensitivity of plant under abiotic stress [14,15,16,17]. The JIP-test elaborated by Strasser and Srivastava [18] is the main explanatory model used to elucidate the changes in fluorescence kinetics. The O-J phase (0–3 ms) describes the gradual reduction of the PSII acceptor side. The J-I phase (3–30 ms) of the transient reflects a reduction of the PQ-pool, and the I-P phase (30–200 ms) is related to the final reduction of electron acceptors in and around PSI: PC, P700 (Photosystem I), the Fe/S (iron-sulfur) clusters and Fd. Analysis of the fluorescence induction curve provides substantial information about the structure and function of the photosynthetic apparatus [17,19]. It has been already utilized in a number of plant species, including cereals, such as barley, under abiotic stresses [15,20,21,22,23,24].

Barley (*Hordeum vulgare* L.) is the fourth most important crop in terms of harvested acreage and production worldwide (FAOSTAT, 2018). Moreover, barley is cultivated globally, well adapted to extreme environments, and thus, it can serve as a model crop for understanding and studying the response to climate change events [25]. Additionally, the assembling of the barley genome sequence and well-established genomic and phenotypic toolkit turned barley into an attractive model species for the studies of monocotyledonous plant biology [26,27].

One of the possible ways to increase barley yield under unfavorable climatic conditions is improving its photosynthetic efficiency under climatic change scenarios. One of the prerequisites is to elucidate in detail the regulatory networks controlling both the construction and the functioning of the photosynthetic machinery. Strikingly, despite the relatively rich dataset regarding the effect of drought stress on photosynthesis and thus plant performance, there are still many open questions regarding the regulation of photosynthesis process [8,21,22,28]. Moreover, the knowledge regarding genes related to the structure and regulation of photosynthesis in barley is not fully covered [21,24,28].

Here, we present a comprehensive study of genetic and physiological dissection of the photosynthesis process in barley under drought stress. Using high throughput transcriptomic data, we were able to identify and annotate almost 150 barley genes crucial for photosynthesis. Moreover, we hunted the possible regulatory interactions between photosynthesis-related genes and transcription factors in barley based on our RNA-Seq data. In addition, we examined the physiological parameters taking advantage of the JIP-test. Using this approach, we indicated photosynthesis as a process that is very early affected in barley seedlings treated with severe drought stress. Given the current attempts in improving photosynthetic efficiency for greater crop yield, the explanation of the genetic basis of that process, together with phenotypic analysis, is significant in terms of both basic studies and potential agricultural application. Furthermore, our results provide a list of candidate genes for future genetic research and improvement of barley drought tolerance by targeting the crucial process-the photosynthesis.

## 2. Results

### 2.1. Transcriptomic Analysis Revealed Expression Changes of Photosynthesis-Related Genes Already Upon the Onset of Drought Stress

**Figure 1:**
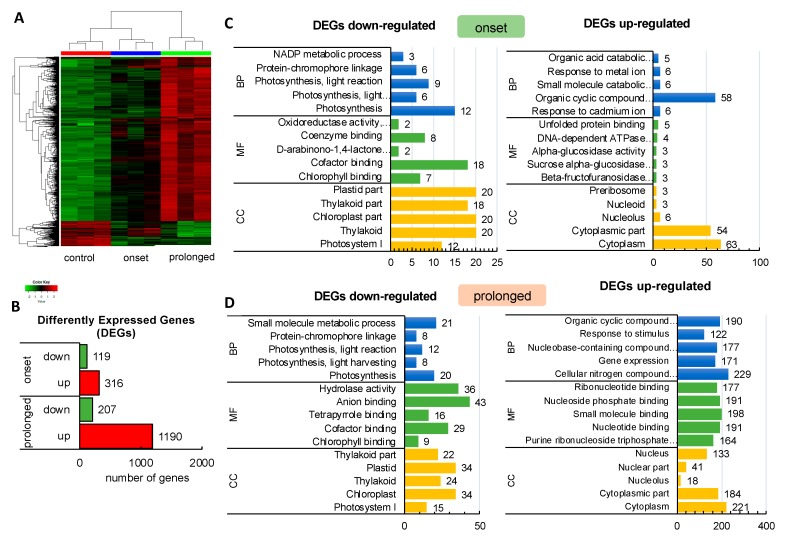
Response of barley transcriptome at the onset and after prolonged drought stress analyzed using Agilent microarray. (**A**) Hierarchical clustering of the genes that were differentially expressed in barley subjected to drought. (**B**) Number of Differently Expressed Genes (DEGs) in response to early (onset) and prolonged drought stress (*p* ≤ 0.01, Fold Change (FC) ≥ 2). (**C**) Gene Ontology enrichment of down- and up-regulated DEGs at the onset of drought stress (*p* ≤ 0.05); BP–biological process, CC–cellular component, MF–molecular function. (**D**) The Gene Ontology enrichment of down- and up-regulated DEGs after prolonged drought stress (*p* ≤ 0.05); BP–biological process, CC–cellular component, MF–molecular function.

First, we employed the microarrays to investigate a leaf transcriptome of barley cv. “Sebastian” subjected to severe water deficit in three time points during the course of the experiment: (1) optimal water supply, before drought stress treatment (10 Days After Sowing (DAS), 14% volumetric water capacity (vwc)); (2) at the onset of drought stress (15 DAS, 3% vwc), and (3) after severe drought stress (25 DAS, 1.5% vwc). The hierarchical clustering of data clearly showed that distinct sets of genes were differentially expressed during the drought stress experiment in barley seedlings (Figure 1A).

The next step was the identification of differentially expressed genes (DEGs). The overall number of high confidence (HC, according to the barley genome version International Barley Sequencing Consortium (IBSC) v2) genes differently regulated under drought conditions in cultivar (cv). “Sebastian” was 1432 (log_2_FC ≥ 2; P ≤ 0.01 after False Discovery Rate (FDR) correction). Then, we split the analysis into the onset of drought stress (vs. control conditions before drought treatment) and prolonged drought (vs. control conditions before drought treatment). The differential analysis of the transcriptome at the onset of drought stress led to the identification of 316 up-regulated and 119 down-regulated HC genes while after the severe drought, 1190 and 207 genes were classified as up- and down-regulated, respectively (Figure 1B).

**Figure 2:**
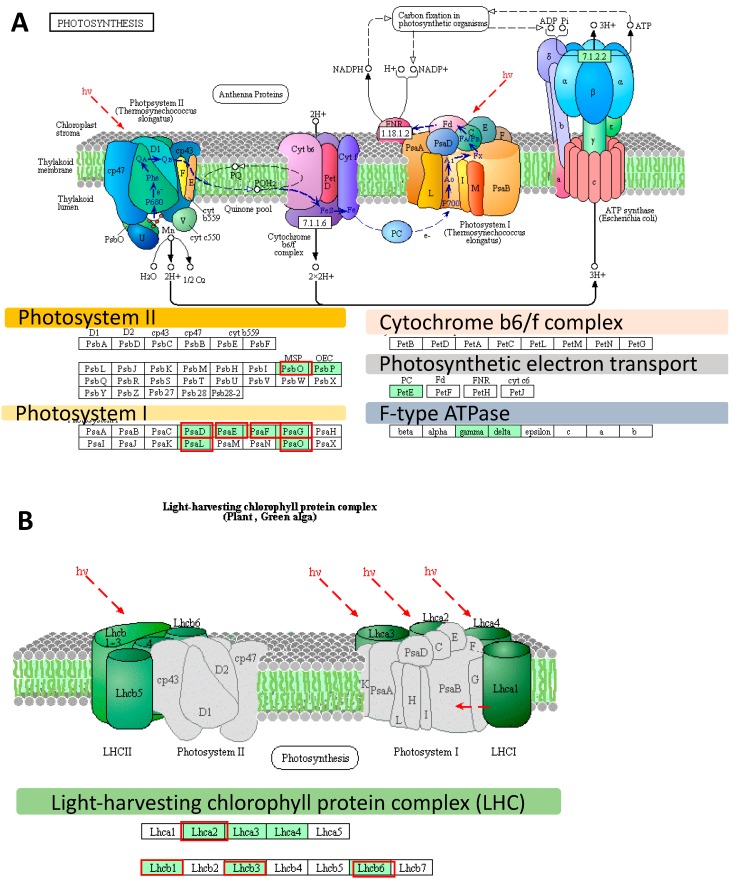
The distribution of differently expressed genes (DEGs) identified using an Agilent microarray in photosynthesis-related pathways, based on the Kyoto Encyclopedia of Genes and Genomes (KEGG) photosynthesis pathway map (http://www.kegg.jp/pathway/map00195). (**A**) Genes mapped using the KEGG as the genes involved in the photosynthesis pathway. (**B**) Genes mapped using the KEGG as encoding the light-harvesting chlorophyll protein complex. The light green boxes indicate the proteins encoded by the Differently Expressed Genes (DEGs). Red frames designate DEGs identified already at the onset of the drought stress. *hν* designates light energy/photons. All genes abbreviations are defined in the Table S1; LHCII—Light-Harvesting Chlorophyll-binding proteins of photosystem II, LHCI—Light-Harvesting Chlorophyll-binding proteins of photosystem I.

Further, we conducted a detailed gene ontology (GO) analysis of all sets of DEGs (enrichment FDR ≤ 0.01). As such, we identified up to five major GO terms in each of the GO categories (BP: Biological Process, CC: Cellular Component, and MF: Molecular Function) (Figure 1C,D). Among the most enriched biological processes associated with down-regulated genes in both sets of DEGs, at the onset and after the prolonged phase of the experiment, were those linked to ‘Photosynthesis’. We focused on genes represented by photosynthesis-related GO terms. Then we filtered out the overlapping records among categories and we extracted the set of genes consisting of 58 DEGs represented by photosynthesis-related GO terms from DEGs at the onset (*n* = 13) and after the prolonged drought stress (*n* = 58, which included the 13 DEGs identified at the drought onset). These genes followed a further detailed investigation using KEGG (The Kyoto Encyclopedia of Genes and Genomes). We undertook pathway reconstruction after KEGG Orthology And Links Annotation (GhostKOALA) annotation of proteins encoded by the set of the abovementioned 58 DEGs. Together, 21 barley genes were mapped using (KEGG) approach as ‘Energy metabolism’ pathway which consisted of ‘Photosynthesis’ (11 DEGs encoding two subunits of PSII, six subunits of PSI, one component of the electron transport chain, two subunits of F-type ATPase), ‘Antenna Proteins’ (eight DEGs encoding three LHCA and three LHCB proteins) and ‘Carbon fixation in photosynthetic organisms’ (two DEGs encoding two enzymes) (Figure 2A,B).

**Figure 3:**
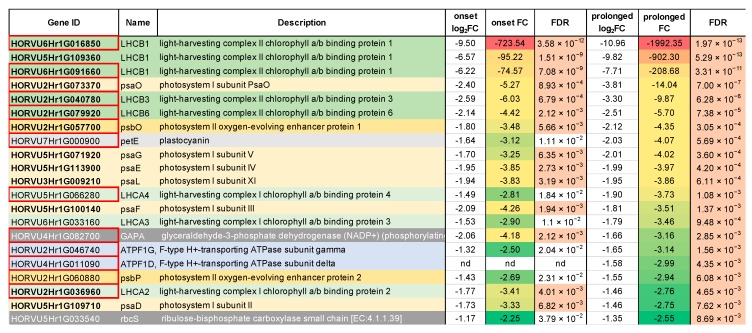
The detailed annotation and expression of fold-changes of DEGs mapped onto the photosynthesis pathway. Red frames in ‘Gene ID’ column designate DEGs already identified at the onset of the drought stress. Green shading indicates genes encoding light-harvesting complex components, yellow indicates subunits of PSI, orange indicates subunits of PSII, gray indicates components of the electron transfer chain, dark gray indicates components of the Calvin cycle. FDR—False Discovery Rate.

The most pronounced changes in expression level were noticed in the case of three genes (HORVU6Hr1G016850, HORVU6Hr1G091660, HORVU5Hr1G109360) encoding the light-harvesting chlorophyll-binding protein 1 (LHCB1) associated with photosystem II (Figure 3). These genes were down-regulated already at the onset of drought stress by 732-, 74-, and 95-fold, and after the prolonged drought, their levels of expression dramatically decreased to 1992-, 208-, and 902-fold, respectively, compared to the control conditions. Interestingly, 13 out of the 21 photosynthesis-related DEGs already responded at the onset of the drought stress (FDR ≤ 0.01). Among them there were genes encoding light harvesting chlorophyll-binding proteins (LHCA2, LHCB1, LHCB3, and LHCB6), photosystem II oxygen-evolving enhancer protein 1 (PsbO), six subunits of photosystem I (psaD, psaE, psaF, psaL, psaG, and psaO) and glyceraldehyde-3-phosphate dehydrogenase (GAPA) (Figure 3).

### 2.2. Deep Transcriptome Sequencing Uncovered a Core Set of Genes Involved in Photosynthesis in Barley

**Figure 4:**
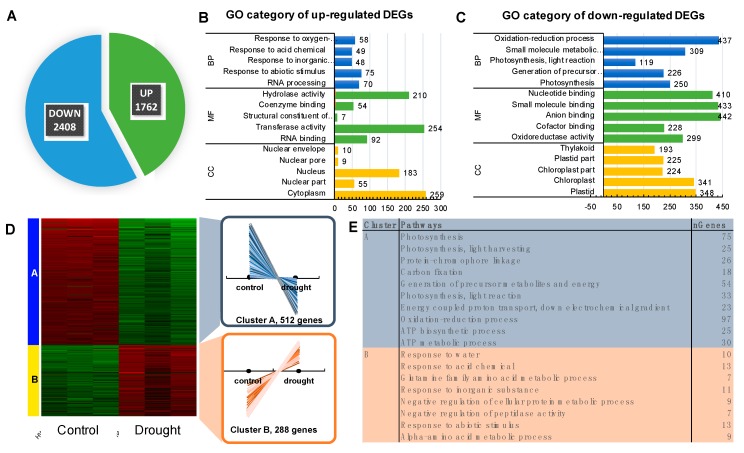
Detailed analysis of the transcriptome response to drought using deep sequencing of the transcriptome (RNA-seq). (**A**) The number of differently regulated genes in response to drought when compared with controls (*p* ≤ 0.01; FC ≥ 2). (**B**) Significantly enriched gene ontology (GO) terms of up-regulated genes (*p* ≤ 0.05); BP–biological process, CC–cellular component, MF–molecular function. (**C**) Significantly enriched gene ontology (GO) terms of down-regulated genes (*p* ≤ 0.05); BP–biological process, CC–cellular component, MF—molecular function. (**D**) The heatmap of the K-means analysis performed using the 1000-top ranked DEGs. (**E**) Significantly enriched GO terms of Biological Processes in each of the two clusters identified. The number of genes downregulated shown in panel A versus panel C and the number of genes up-regulated presented in panel A versus B differs since only the five most significant GO terms are depicted for each GO category at panels B and C, thus not all genes are represented.

Taking into account that Agilent Barley Gene Expression Microarray represents ca. 30% of barley genes, we undertook deep sequencing of the transcriptome aiming at the identification of a core set of genes associated, not only with the photosystem structure, but also with the regulation of the photosynthesis process under drought stress. We used the same experimental design, but we investigated only two timepoints: 10 and 25 DAS. A total of more than 40 million high quality 125-bp paired-end reads were generated by RNA-Seq for each sample. To focus on the relevant drought-responsive genes, we considered a gene to be differentially expressed when its FDR was less than 1%, and its log_2_FC was a minimum of two. Using these criteria, we identified 1762 up- and 2408 down-regulated genes after prolonged drought stress when compared to control conditions before drought treatment (Figure 4A). We subjected each set of DEGs to a functional enrichment analysis (Enrichment FDR ≤ 0.01). As such, we identified up to five major GO terms in each of the GO categories. Among the most over-represented biological processes (BPs) of the up-regulated genes, we found ‘Response to abiotic stimulus’ and ‘RNA processing’ (Figure 4B, Table S2), whereas in the case of down-regulated genes, the most significant BPs were those associated with ‘Photosynthesis’ (Figure 4C).

Further, we employed K-means clustering of 1000 topmost variable genes ranked by the standard deviation. We identified two clusters of DEGs based on their pattern of expression (Figure 4D). The most significant GO terms in the BP category in Cluster A were related to photosynthesis, whereas in Cluster B were associated with ‘response to abiotic stimulus’, among others. As expected, we observed the opposite tendency in expression among the genes classified in both clusters: in Cluster A, the significant downregulation in response to drought was observed, whereas genes categorized in Cluster B demonstrated up-regulation triggered by water deficit (Figure 4D,E).

**Figure 5:**
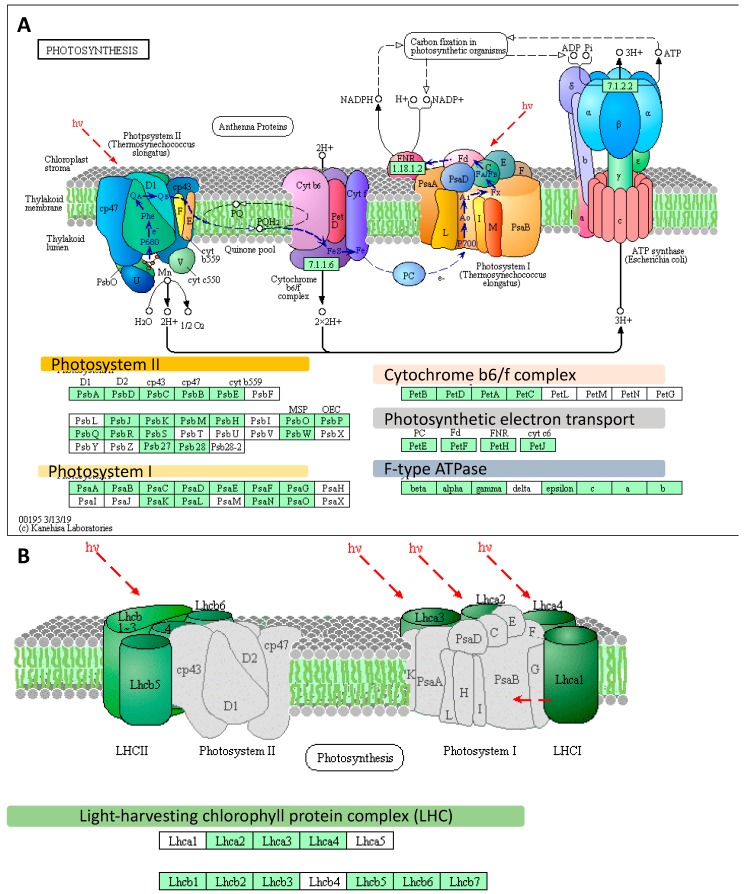
The distribution of differently expressed genes (DEGs) identified using RNA-seq in photosynthesis-related pathways, based on the KEGG photosynthesis pathway map (http://www.kegg.jp/pathway/map00195). (**A**) DEGs represented in the photosynthesis pathway. (**B**) Genes mapped using the KEGG as those encoding the light-harvesting chlorophyll-protein complex. The light green boxes indicate the proteins encoded DEGs; *hν* designates light energy/photons. All genes abbreviations are defined in the Table S1; LHCII—Light-Harvesting Complexes of Photosystem II, LHCI—Light-Harvesting Complexes of Photosystem I.

We extracted genes represented by the following GO terms: Photosynthesis; Photosynthesis, light-harvesting; Photosynthesis, light reaction; Generation of precursor metabolites and energy, and Carbon fixation. After filtering that allowed to remove the genes with redundant values, we used a set of 191 DEGs for further examination with the use of KEGG Pathway Reconstruction, following the same strategy as the above-mentioned in the case of microarray data. The most enriched pathway in terms of the number of annotated genes (n = 147) was ‘Energy metabolism’ pathway that consisted of ‘Photosynthesis’ (93 DEGs) encoding 16 subunits of PSII (34 DEGs), 10 subunits of PSI (20 DEGs), four components of the electron transport chain (seven DEGs), four components of cytochrome b6/f complex (10 DEGs), nine subunits of ATPase (22 DEGs)) (Figure 5A), ‘Antenna Proteins’ (21 DEGs encoding four LHCA and five LHCB proteins) (Figure 5B), and ‘Carbon fixation in photosynthetic organisms’ (33 DEGs encoding 18 enzymes) (Table S1). Remarkably, among the top 20 genes ranked based on their highest changes in expression level were those encoding LHCB1 (light-harvesting complex II chlorophyll a/b binding protein 1), rbsS (ribulose-bisphosphate carboxylase small chain [EC:4.1.1.39]), PsbQ (photosystem II oxygen-evolving enhancer protein 3), psaO (photosystem I subunit), and petF (ferredoxin).

### 2.3. Promoter Analysis Coupled with Transcription Factors (TFs) Identified in the Transcriptomic Study Predicts New Candidates for Further Studies

**Figure 6:**
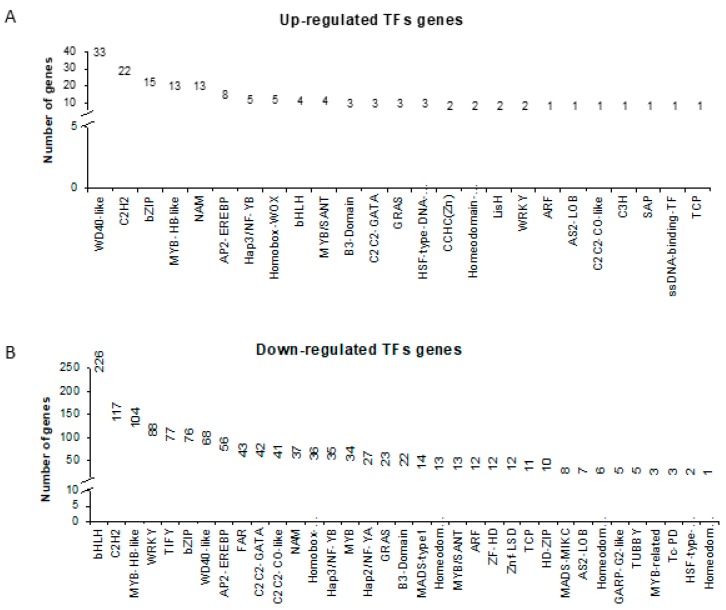
Transcription factor families in DEGs dataset. (**A**) Transcription Factor (TF) families and the number of genes encoding the members of each family within the set of up-regulated genes. (**B**) TF families and the number of genes encoding the members of each family within the set of down-regulated genes.

Within all DEGs identified in our study, 1438 (34% of all DEGs) encoded transcription factors, 149 of which were up- and 1289 were down-regulated. The highest number of genes were found within the following TF families: WD40-like (33), C2H2 (22), bZIP (15), MYB-HB-like (13), NAC/NAM (13) (Fig. 6A) when up-regulated genes were considered, and bHLH (226), C2H2 (117), MYB-HB-like (104), WRKY (88) and TIFY (77) in the down-regulated DEG set (Figure 6B). We hypothesized that detailed analysis of identified TFs in both up- and down-regulated sets of DEGs together with the promoter analysis of photosynthesis-related genes will provide novel insight into understanding the regulation of photosynthesis under drought by the designation of potential regulatory pairs ‘TF and a target gene’. Moreover, we presumed that some of the photosynthesis-related co-expressed genes (*n* = 147) might share cis-regulatory sequences. Therefore, their upstream sequences were subjected to motif discovery analysis. Together, 15119 binding sites for 218 TFs were found in 147 promoter sequences of photosynthesis-related genes. Next, we checked whether TFs identified in our approach (149 and 1289 in up- and down-regulated dataset) were among those predicted to bind to the discovered regulatory motifs in upstream sequences of photosynthesis-related genes. As such, we identified 17 genes encoding TFs characterized by a changed expression in response to drought stress (eight and nine within the up- and down-regulated DEGs set, respectively) with predicted binding sites within the promoter region of photosynthesis-related genes (Table 1). Among genes encoding TFs, the most represented group was bZIP (*n* = 6) and MYB-related (*n* = 3) transcription factor families. Using this approach, we suggest 17 putative regulatory pairs between TFs and their putative targets within photosynthesis-related genes (Table 1; Table S3). Designated as potential targets were genes encoding subunits of PSI, PSII, electron transfer chain, as well as Calvin cycle components. These results can serve as a starting point for a more advanced study aimed at the elucidation of photosynthesis regulation at the molecular level during drought stress in barley.

### 2.4. Physiological Analyses Showed a Pronounced Decline of Photosynthesis Performance only after Prolonged Drought Stress

To further explore the response of barley photosynthetic apparatus in cultivar “Sebastian” under the drought stress, we measured selected physiological parameters linked to photosynthesis. Together with transcriptome data, these results gave us a better overview of the physiological state of stressed barley plants. It also enabled us to characterize the earliest changes in plants responding to the water deficit in terms of photosynthesis.

**Figure 7:**
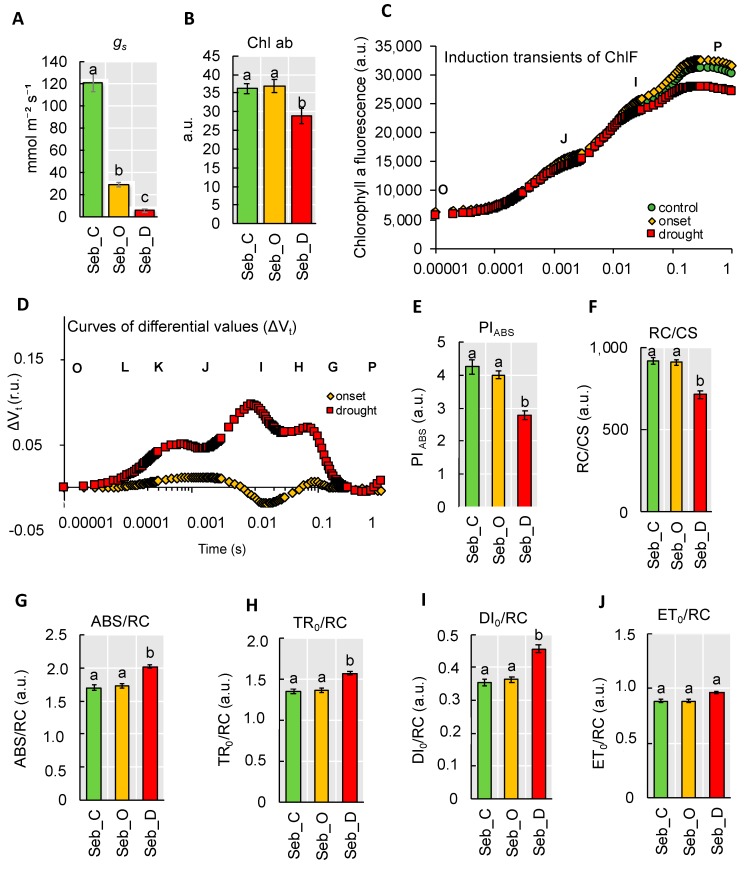
Physiological analysis of barley seedlings exposed to drought stress. (**A**) The stomatal conductance (g_s_); (**B**) chlorophyll index; (**C**) The OJIP curve; (**D**) The relative variable fluorescence (ΔVt); (**E**) Performance index (PI_ABS_); (**F**) Active reaction centers per illuminated cross-section (RC/CS); (**G**) Absorption flux energy per active reaction center (ABS/RC); (**H**) Trapped flux of energy in Photosystem (PS) per Reaction Centre (RC) (TR/RC); (**I**) Dissipated energy flux per RC (DI/RC); (**J**) Electron transport per RC (ET/RC). Each of the values presented is the mean ± SE, the mean was calculated from three biological replicates each replicate comprised three plants. r.u., relative units. Different lower-case letters indicate the statistical differences between conditions according to the two-way ANOVA followed by Tukey Honestly Significant Differences (HSD) test (*p* < 0.05). Seb_C—control, Seb_O—onset of drought stress, Seb_D—prolonged drought stress. a.u.—arbitrary units.

Taking into account that the first and most rapid physiological reaction to water deficit is stomatal closure, we measured stomatal conductance under control (C), at the onset of drought stress (O) and after prolonged drought stress (D). We noticed that at the onset of drought stress, the stomata were already significantly closed, and the value of the stomatal conductance dropped significantly till the end of the experiment (Figure 7A). Although the stomatal closure is considered as a water-saving mechanism, it also leads to photoinhibition due to the negative impact on gas exchange. Decrease in chlorophyll (Chl) content, the main photosynthetic pigment, is a commonly observed phenomenon under drought stress. Interestingly, at the onset of drought stress, there was no significant change in Chl content noted, whereas, after 10 days of water deficit, it dropped more than 20% when compared to the control (Figure 7B). Next, to investigate the photosynthetic efficiency of barley plants under drought stress, we employed a JIP-test based on chlorophyll *a* fluorescence measurements. It allowed us to monitor mainly the light phase of photosynthesis in leaves from exciton migration between antennae pigments of neighboring photosynthetic units and the electron transfer reactions at the donor and acceptor sites of Photosystem II (PSII) and Photosystem I (PSI) and finally between both of them. The OJIP chlorophyll induction curves have three main phases: O-J, J-I, and I-P. The O-J phase denotes the gradual reduction of Q_A_, the primary electron acceptor in PSII. The J-I phase is responsible for chlorophyll FL quenching, which characterizes the activity of the oxygen-evolving complex (OEC) on the donor side of PSII. The IP-kinetics may give information on effects on PSI activity or at least on the PSI activity relative to PSII activity [19]. We observed that the maximal level of fluorescence (F_M_, denoted as P in the Figure 7C) declined only after a prolonged drought. Differential curves, ΔV_t_, resulting from a subtraction of parameter’s values of the control plants from the records at the onset and after prolonged drought stress, respectively allowed us to notice that water deficit at each of the studied phase of experiment differently affected photosynthesis in barley leaves (Figure 7D). After prolonged drought stress, the positive ΔK-band (0.0003 s) was noted. The ΔK-band appears in response to stress and points to disturbances of the water diffusion process, and distracting electron balance between OEC (oxygen-evolving complex) and tyrosine.

The OJIP transients were translated into biophysical parameters [18]—phenomenological energy fluxes per illuminated leaf area (cross-section; CS); quantum yields (φP_0_, φE_0_, ΨE_0_, φR_0,_ and δR_0_); specific activities per reaction centers (RC) and performance index (PI_ABS_) (Table 2).

First, we looked closer to PI_ABS_ value since it contained information about three independent parameters: density of reaction centers, which corresponds to the absorption flux, quantum yield of trapping and probability that a trapped exciton will move an electron into electron transport chain beyond Q_A_. This parameter was significantly decreased only after prolonged drought stress by 35% (Figure 7E). A similar tendency of changes was observed in the case of a number of active reaction centers per illuminated cross-section (RC/CS) that reduced to 70% of control after drought (Figure 7F). In case of parameters per active reaction center (RC) such as the absorbed light energy per unit reaction center (ABS/RC), the absorbed light energy used for reduction of Q_A_ per RC (TR_0_/RC), and the dissipated energy per RC (DI_o_/RC) (Figure 7G–I) marked increase was also noticed only after prolonged drought stress. No significant changes in relation to control conditions were detected for the absorbed light energy used for electron transfer per RC (ET_o_/RC) (Figure 7J). In order to track the energy fluxes through photosystem at the quantum level, we analyzed changes in values of φP_o_ that is denoted as primary PSII photochemistry related to photon trapping, and parameters describing electron transfer from Q_A_ to intersystem electron acceptors (ψE_o_) and parameters describing electron transport to the final PSI electron acceptors (δR_o_). We noted a significant reduction in parameters linked with φP_0_ and electron transport only in response to prolonged drought (Table S4), which is in line with observations of OJIP curve shapes.

### 2.5. Drought Stress Influenced Barley Photosynthesis in a Universal, Genotype-Independent Manner

**Figure 8:**
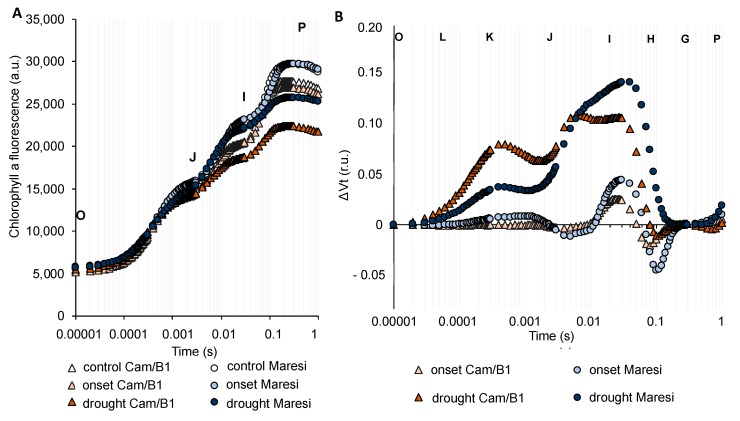
Chlorophyll *a* fluorescence induction curves of the “Cam/B1” and “Maresi” genotypes exposed to the onset and prolonged drought stress. (**A**) The effects of drought stress on the OJIP transients of the genotypes studied. (**B**) The effects of drought stress on the relative variable fluorescence (ΔVt) of the genotypes studied. Values are means (*n* = 15). r.u.—relative units; a.u.—arbitrary units.

Next, we addressed a question of whether the physiological response to drought stress in terms of photosynthesis in barley is universal or cultivar-dependent. For this reason, we undertook a chlorophyll *a* fluorescence analysis during drought stress using two genetically distant barley cultivars –“Maresi”–the European cv. described as drought-sensitive and Syrian “Cam/B1” cv. tolerant to drought stress [28,29]. The drought stress was performed according to the same protocol as in the case of “Sebastian” cv. Our data showed that both, “Maresi” and “Cam/B1”, exhibited a much more pronounced reaction of photosynthetic apparatus in response to drought conditions when compared with “Sebastian”. The analysis of OJIP transients of both cultivars indicated that the I-P part of the OJIP transient was decreased in response to prolonged drought stress. Moreover, it was clearly visible that the decreased maximal level of fluorescence (F_M_, denoted as P in Figure 8A) occurred only after prolonged drought. With the aim of better visualizing the effect of drought stress on the transient dynamics in the two genotypes tested, the curves were plotted as relative variable fluorescence, ΔVt. Here, we noted that the onset of drought stress impacted only on the ΔI-ΔP part of the induction curve (but not significantly), whereas the prolonged drought treatment disrupted both parts of the OJIP transient (Figure 8B). It was clearly visible the ΔK-band together with the presence of significant ΔI bands, with shoulders to ΔH observed for both genotypes (Figure 8B). It was in line with results obtained in the case of “Sebastian”. Our analysis of changes in the value of PI_ABS_ in “Maresi” and “Cam/B1” also showed the same tendency as in the case of “Sebastian”. PI_ABS_ harshly declined only after prolonged stress treatment (Table S4). The largest impact of prolonged drought on barley photosynthesis performance was also confirmed by the analyses of energy fluxes per unit of the active reaction center (RC), per illuminated cross-section (CS), or when quantum efficiencies of photosynthetic performance were examined for these two cultivars (Table S4).

### 2.6. Rapid Dehydration Disturbed the Balance in Photosynthesis Reactions Probably due to the Disintegration of Components Protecting the Oxygen Evolving Complex

**Figure 9:**
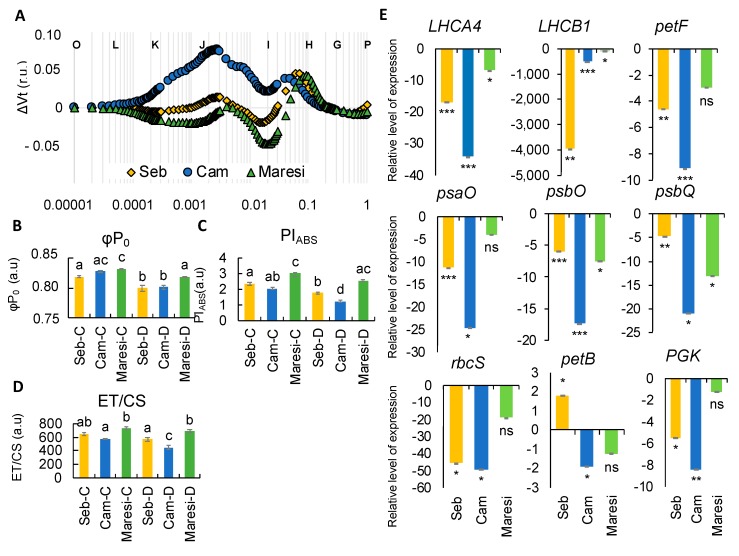
The analysis of barley seedlings response to rapid dehydration. (**A**) The effects of rapid dehydration on the relative variable fluorescence (ΔVt) of the genotypes studied. Values are means (*n* = 15). r.u.—Relative units. (**B**) Maximum quantum yield of primary photochemistry (ϕP_0_). (**C**) Performance Index (PI_ABS_). (**D**) Electron transport per illuminated cross-section (ET/CS). (**E**) Analysis of the expression of selected genes encoding components of the photosynthetic machinery. r.u.—Relative units. Different lower-case letters indicate the statistical differences between conditions according to the two-way ANOVA followed by the Tukey HSD test (*p* < 0.05). In case of genes expression, asterisks indicate the statistically significant changes of expression after dehydration when compared with controls in the case of each genotype (* *p* ≤ 0.05, ** *p* ≤ 0.01, *** *p* ≤ 0.001). a.u.—arbitrary units.

Taking into account that plants encounter rapid dehydration events during the life cycle, we applied rapid dehydration shock to all of the above-mentioned barley cultivars, “Sebastian”, “Cam/B1”, and “Maresi”. Concomitantly with the examination of photosynthesis at the physiological level, we also tested the expression of several genes selected from the transcriptome sequencing experiment (Figure 9). It is worth noting and important that rapid dehydration evoked a similar pattern of OJIP curve as early drought stress, mostly affecting the ΔI-ΔP phase. Only “Cam/B1” exhibited ΔK-band together with the ΔJ-ΔI shoulder that was absent in the case of ‘Sebastian’ and “Maresi”. It can be concluded that in response to rapid dehydration in “Cam/B1”, the balance of electron transport is disturbed. On the other side only in “Sebastian” and “Maresi”, the negative ΔI-band was observed (Figure 9A). Despite the fact that changes in most of the parameters were rather subtle, we observed a significant decrease in maximum quantum yield of the primary PSII photochemistry (φP_0_), performance index PI_ABS_ and the electron transport measured per illuminated cross-section (ET/CS) (Figure 9B–D). In each case, the most detrimental effect was noticed in “Cam/B1”. All of these are consistent with the shape of OJIP curves plotted after rapid dehydration. Strikingly, the analysis of the expression of several photosynthesis-related genes revealed another similarity with the onset of drought stress applied during the growth in soil. First, in all genotypes tested we noticed a significant downregulation of genes encoding light-harvesting chlorophyll-binding proteins such as LHCB1 (HORVU1HR1G088900) and LHCA4 (HORVU5HR1G066280) but also PsaO (HORVU2HR1G073370) encoding subunit of PSI and PsbO (HORVU2HR1G057700) which is a component of oxygen-evolving complex (OEC) (Figure 9E). Moreover, the transcription of the above-mentioned genes was most affected in the case of ‘Cam/B1’ (except for *LHCB1* encoded by HORVU1HR1G088900). Especially, the down-regulation of *PsbO* (HORVU2HR1G057700), *PsbQ* (HORVU6HR1G051650), can be linked with the appearance of the ΔK-band when the ‘Cam/B1’ fluorescence curve was plotted. It suggests the negative effect of the rapid dehydration on the OEC function.

### 2.7. Exogenously Applied Abscisic Acid (ABA) Mostly Affected the Electron Transport in Barley

**Figure 10:**
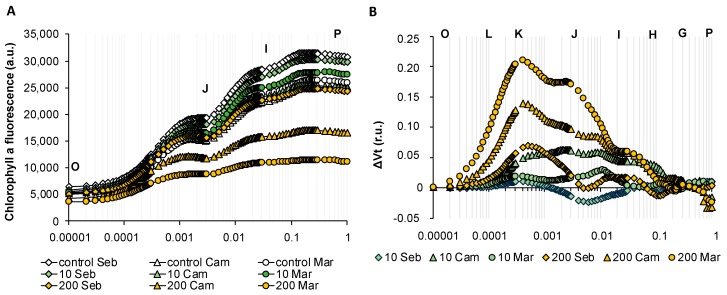
Chlorophyll *a* fluorescence induction curves of barley in response to different Abscisic Acid (ABA) concentrations during early seedling development. (**A**) The effects of ABA on the OJIP transients of the genotypes studied. (**B**) The effects of ABA on the relative variable fluorescence (ΔVt) of the genotypes studied. Values are means (*n* = 15). r.u.—Relative units.

Taking into account that ABA plays a crucial role in drought stress response, we asked whether and how ABA implied photosynthesis. Surprisingly, the reaction of all genotypes tested was profoundly different on the photosynthesis level when compared with changes observed after drought treatment. Although subtle differences among cultivars studied were indicated, the tendency of alterations evoked by ABA was similar. We applied two ABA concentrations that provoked mild (10 µM) and strong (200 µM) reactions, respectively, during seedlings development in terms of growth inhibition as per our earlier studies [30]. The first applied ABA concentration (10 µM) did not reveal significant changes in OJIP curves, whereas the application of higher ABA concentration (200 µM) triggered a decrease of maximal fluorescence value (F_M_ = P) in all of the genotypes tested. It should be underlined that the treatment with 200 µM ABA enabled us also to distinguish among genotypes since “Maresi” and “Cam/B1” displayed much more distracted OJIP transient than “Sebastian” (Figure 10A). Next, we analyzed ABA-dependent changes in more detail plotting curves as relative variable fluorescence, ΔV_t_ (Figure 10B). The overall tendency of the reaction of the barley photosynthetic apparatus to 200 µM ABA was displayed mainly by a positive ΔK-band and a shoulder in the ΔJ-ΔI part of the curve. Therefore, we suggest that ABA mainly affects the excitation energy fate between thermal dissipation in the photosynthetic antenna absorption and primary photochemical reaction in the reaction center when young seedlings were treated. Remarkably, the main difference between the picture drawn for a response to 200 µM ABA and prolonged drought stress is the lack of changes in the I-P part of the transients when ABA response was analyzed. The performance index PI_ABS_ was reduced in all genotypes tested in response to 200 uM ABA. In the lower ABA concentration - 10 µM ABA, PI_ABS_ was reduced only in “Maresi” and “Cam/B1”. This result corresponds with the decreased value of F_M_ observed when OJIP transients were analyzed. The higher concentration of ABA significantly affected the quantum yields of electron fate such as ϕE0, ψE0, and ϕR0 which are descriptors of electron transfer between PSII acceptor and donor side and beyond PSII. The energy fluxes per cross-section (CS), such as ABS/CS and RC/CS, and quantum efficiencies of photosynthetic performance such as φP_0_ were decreased to a greater extent in response to higher ABA concentration. It indicated that the higher ABA concentration—200 µM had a stronger effect on the photosynthetic activity in barley (Table S5).

## 3. Discussion

Balanced photosynthesis under abiotic stress conditions, such as drought, is essential for better survival and also agricultural benefits in terms of biomass and yield [31]. The aim of this study was to uncover the molecular basis of the photosynthesis process under drought stress in barley. To address that goal we: (i) conducted transcriptomic examination (microarray and RNA-seq) aimed at identification of barley genes encoding core-components of photosynthetic machinery and the potential regulators of the process; furthermore, we (ii) performed detailed photosynthesis analysis using OJIP test and focusing on the exact pattern of changes in response to drought, rapid dehydration, and abscisic acid. Rather than focusing on individual genes, our strategy was pointed to the identification of groups of genes with similar expression patterns. As such, we identified and annotated almost 150 barley genes as crucial core-components of photosystems, electron transport components, and Calvin cycle enzymes.

Here, we mainly described the effect of drought stress in regard to the light phase of photosynthesis since the OJIP-test enabled us to track changes in that part of the process. However, RNA-seq analysis allowed us also to identify several genes involved in the dark phase of photosynthesis. Complexes of PSII and PSI take part in light absorption, electron transport, and further electron conversion. These photosystems basically comprise: (i) antenna systems consisting of light-harvesting complexes (LHC) that increase the capacity of light absorption and contribute to photoprotection, and (ii) reaction centers (RC) that convert light energy into chemical energy. Members of LHC are associated with PSI (LHCA proteins) and PSII (LHCB proteins) [32,33]. In plants, more than 60% of all chlorophyll is bound to light-harvesting complexes [34,35]. Water deficit led to a significant reduction in chlorophyll content in barley, which correlated positively with the changes in absorption energy per cross-section (ABS/CS) during the course of drought experiment (Figure S1). The light absorbed by chlorophyll can be utilized in photochemistry, the excess of energy can be dissipated as heat, or it can be re-emitted as a chlorophyll fluorescence [36]. Since the PI_ABS_ (Performance Index) considers the three main steps that regulate photosynthetic activity by a PSII reaction center (RC) complex, precisely (1) the absorption of light energy (ABS), (2) trapping of excitation energy (TR), and (3) conversion of excitation energy to electron transport (ET), we used it in order to evaluate the general photosynthetic activity of barley plants under drought. Moreover, PI_ABS_ was already pointed as very sensitive to drought conditions in monocots [21,22,29,37,38]. In our study, in the case of each genotype tested, PI_ABS_ was significantly reduced only after prolonged drought stress to 55–70% of the control, depending on the genotype (Table S4). Moreover, we observed a strong correlation between the PI_ABS_ and descriptors of electron transport such as (1) quantum yield for electron transport from Q_A_^−^ to plastoquinone (ϕE0; *R*^2^ = 0.95; Table S6), (2) the probability (at time 0) that a trapped exciton moves an electron into the electron transport chain beyond Q_A_^–^(ψE0; *R*^2^ = 0.76; Table S6), (3) a quantum yield for the reduction of the end electron acceptors at the PSI acceptor side (ϕR0; *R*^2^ = 0.77; Table S6), and (4) the probability with which an electron from the intersystem electron carriers will move to reduce the end acceptors at the PSI acceptor side (δR0; *R*^2^ = 0.63; Table S6). These results clearly demonstrate the strong link between analyzed features in response to drought stress.

Another parameter highly correlated with PI_ABS_ was the number of active reaction centers per illuminated area (RC/CS; *R*^2^ = 0.91; Table S6). We found on average the 20% reduction of active reaction centers of PSII expressed as RC/CS (the number of active reaction centers per unit of illuminated area) in drought-exposed “Sebastian” and “Maresi”, while at the same conditions in “Cam/B1” almost 45% of RC/CS were inactivated (Table S4). As a consequence, it led to changes in relative antenna size linked to active RC (ABS/RC), which increased by 10–30% in barley cultivars under drought stress depending on the genotype (Table S4). Again, in the case of “Cam/B1”, we noticed the most pronounced effect. As expected, this parameter was negatively correlated with RC/CS (*R*^2^ = -0.84), but also the same trend of correlation was observed with PI_ABS_ (*R*^2^ = -0.93; Table S6). Since the excess of energy unutilized in photochemistry is dissipated as heat, the increase of DI_0_/RC (dissipated energy per active RC) was expected. Indeed, the value of this parameter raised depending on the genotype in a range to 130–160% of the control (Table S4). Together, these results clearly pointed to the high impairment of photosynthesis efficiency in barley exposed to drought, regardless of the genetic background. However, differences between genotypes were also noted. In previous studies with the use of “Cam/B1” and “Maresi”, it was demonstrated that ‘Cam/B1’ has a drought-tolerant phenotype when compared with “Maresi” [29,39,40]. Surprisingly, in the presented study we found the most harmful effect on photosynthesis-related parameters in ‘Cam/B1’ genotype. It is worth to note that our experiments and the above-mentioned studies differed in regard to drought stress treatments and that the stress applied in our study was the most severe. Thus, it is not surprising that the response of the genotypes studied was faster, and therefore, might be different when compared to the previously published research. Filek et al. demonstrated that at the beginning of stress treatment, “Cam/B1” performed worse than “Maresi” when PSII efficiency was determined using F_V_/F_M_ parameter, describing the maximum of photochemical efficiency of PSII. However, this tendency changed after 10-days of drought treatment and better adaptation of “Cam/B1” than “Maresi” to drought stress was demonstrated [29]. It is worth noting that a drought-tolerant phenotype of ‘Cam/B1’ is dependent on a better water-saving mechanism that was manifested mainly by a higher Relative Water Content (RWC), higher sugar content, better hydrated Mn (II) ion, lower content of Fd (III) ions when compared to “Maresi” under drought stress [29,40]. Moreover, chloroplasts of “Cam/B1” were shown to have a larger surface area and less degradation of their structure during drought stress in comparison to “Maresi”. Also, “Cam/B1” was characterized by a greater accumulation of carotenoids in chloroplasts [40]. Another study, focusing on metabolomics, demonstrated better metabolomic protection of leaves in “Cam/B1” than in “Maresi” mainly in terms of phenolic metabolites accumulation [41]. Secondary phenolic metabolites, including flavonoid glycoconjugates, were reported to have a protective effect in chloroplasts, the cytoplasm, and the nucleus. In addition, the earliness of “Cam/B1” noted in the previous studies can affect the physiological response to drought stress [42]. It is associated with a phenomenon of drought escape strategy, which ensures completion of a lifecycle before the environmental conditions become seriously unfavorable. In addition, it was also showed that “Cam/B1” is able to activate the transcription of crucial stress-responsive genes (such as encoding transcription factors from NAC (NAM, ATAF, CUC), bHLH (Helix Loop Helix), or TGA (Teosinte Glume Architecture) families, proteins involved in energy generation, cytoskeleton formation, cellular signaling, or drought escape) faster than the drought-sensitive “Maresi” [28]. These results together clearly showed that the drought-tolerant phenotype of “Cam/B1” is based rather on water-saving mechanisms preventing dehydration rather than photosynthesis efficiency under stress, which is in line with our observations.

The aforementioned lower light absorption expressed by reduced ABS/CS that in turn results in changes in energy trapping and its further transport, and thus, less efficient photosynthesis can be highly related to downregulation of photosynthetic machinery components. Our transcriptomic analysis revealed 114 DEGs related to the light phase of photosynthesis, including 24 DEGs encoding PSI and 51 DEGs encoding PSII components that were downregulated in response to water deficits. Among these downregulated DEGs, 13 encode light-harvesting chlorophyll-binding proteins such as LHCB1, and one encodes LHCB2, LHCB3, LHCB, and LHCB7, respectively (Table S1). It is worth noting that in our study, genes for LHCB1 (HORVU6Hr1G091650, FC = -3743; HORVU1Hr1G078380, FC = -2485) were the most downregulated in a whole dataset in response to drought demonstrating their stress sensitivity (Table S1). Moreover, LHCB1 and LHCB2 proteins are by far the most abundant, and thus, crucial for photosynthesis in plants [35]. In Arabidopsis, it was shown that the lack of both resulted in reduced light absorption, while in the absence of the LHCB5 and LHCB6 plants were characterized by the decreased efficiency of energy transfer from LHCII to reaction centers of PSII [35]. These results are in line with the reduction in values of chlorophyll fluorescence parameters in our study that further may be linked to reduced transcription activity of the above-mentioned genes. Furthermore, we identified four DEGs encoding light-harvesting chlorophyll-binding proteins associated with PSI—LHCA2, LHCA3, and LHCA4. The light-harvesting antenna of PSI forms dimers *LHCA1/LHCA4* and *LHCA3*/*LHCA2*. Interestingly, it was demonstrated that the absence of one of the dimer components leaves a hole that cannot be replaced by other LHCA protein [43]. Hence, it leads to decreased light absorption that was observed in our study based on the OJIP test.

Generally, the dynamics of the fluorescence induction transient may be analyzed by plotting the curves at the time of 1 s on a logarithmic scale. Stress factors harshly modify the shapes of the differential curves, and the induced changes are designated by the appearance of specific bands visible when plots are generated [44]. In the case of prolonged drought stress, we noted the appearance of the ΔK (at 300 µs) band in all genotypes tested. It may be related to the decrease of the oxygen-evolving complex (OEC) activity [14]. OEC is considered as one of the most sensitive components in the photosynthetic electron transport chain [14]. Its diminished performance is usually caused by an electron transport disorder, impairment of which was also observed in our studies when quantum efficiencies were investigated (Table S4). On the lumen side of the PSII complex close to the D1 reaction center subunit (encoded by *psbA* gene), the extrinsic proteins PsbO, PsbP, and PsbQ are located, which form a ‘cap’ over the oxygen-evolving center (OEC) that splits the water into molecular oxygen, electrons, and protons [45]. The PsbO subunit is critical for stabilizing the Mn cluster and has been suggested to form a hydrophilic “pore” connecting the OEC with the lumenal surface [46]. Moreover, PsbO is attached to the PSII core via the large extrinsic loops of chlorophyll apoproteins such as PsbC (CP43) and PsbE (CP47) [46]. In our study, we were able to annotate genes encoding all of the above-mentioned proteins, and all were downregulated under drought stress in barley. Probably, it might be the effect of lower light absorption or oxidative stress implied by drought on PSII. The fluorescence curve plots recorded after prolonged drought in all cultivars tested exhibited a high ∆K-band (Figure 7). We also observed a significant downregulation of genes encoding PsbO (HORVU2Hr1G057700; FC = −43), PsbP (HORVU2Hr1G060880, FC = −35; HORVU4Hr1G000230, FC = −9), and PsbQ (HORVU2Hr1G043960, FC = −72; HORVU2Hr1G080260, FC = −7; HORVU6Hr1G051650, FC = −202). It is worth noting that in *Xerophyta humilis* under drought stress, the relation between the decrease in *PsbO* and *PsbP* mRNA levels was associated with a decline at the protein levels [47]. Moreover, the level of *PsbC* (HORVU2Hr1G062030, FC = −4) and *PsbE* (HORVU5Hr1G065050, FC =; HORVU6Hr1G049390, FC = −5.6), that play a significant role in attaching *PsbO* to OEC was also decreased in response to drought in our study.

Among 4170 DEGs, we identified 1438 TFs (34% of DEGs) that were either up- or down-regulated. In both these categories, the five top TFs families included members of the WD40-like, bZIP, MYB-HB-like, and bZIP factors. Our examination of the promoter region of 147 photosynthesis-related barley genes enabled us to link differently regulated TFs with transcription factor binding sites within the upstream region in several key photosynthetic genes. When taking a closer look at these associations, we found that there is a relation between GOLDEN2-like (G2-like) TF (encoded by HORVU6Hr1G019650, FC = −7.20) and one of the most downregulated genes encoding LHCB1 (HORVU5Hr1G109260, FC = −1357.64). G2-like transcription factors, firstly identified in maize [48], have two domains, including a Myb-DNA binding domain (DBD, containing an HLH motif) and a C-terminal domain (containing a conserved GCT box). Strikingly, it was already demonstrated in Arabidopsis that double mutants *glk1 glk2* were pale green in all photosynthetic tissues and showed reduced grana thylakoids in chloroplasts [49]. Moreover, the overexpression of G2-like in rice enhanced the transcription of a set of nucleus-encoded genes related to chloroplast functions and plastid-encoded genes [50,51]. In our study, both G2-like TFs and their potential targets *LHCB1* and ATPF0B (F-type H+-transporting ATPase subunit b) were downregulated, probably in response to cellular events related to drought stress (Table S3). Remarkably, this interaction seems to have more links with other photosynthetically-related components, as recent findings demonstrated [52]. In Arabidopsis, G2-like TFs are engaged in ABA signaling and directly associates with the promoter region of *WRKY40,* which is another TF important for stress transduction [52]. The expression of *LHCB2* was directly regulated by WRKY40 [53]. In our study, we also identified the gene encoding the putative ortholog of *AtWRKY33* (HORVU3Hr1G088200, FC = −3.78; (Table S3)) as potentially engaged in the regulation of *LHCB1* (HORVU5Hr1G087250, FC = −13.1; (Table S3)) expression. Interestingly, in Arabidopsis exposed to heat stress, the repression of *AtWRKY33* was observed. The *wrky33* mutants were characterized by inhibited seed germination, lower survival, and electrolytic leakage under heat stress. On the contrary, increased tolerance towards heat stress was recorded in line with overexpressing *AtWRKY33* [54]. Taking into account the proved positive role of WRKY33 in transcription regulation, we postulate that its down-regulation in barley exposed to drought stress may impact the inability to regulate its targets positively. Therefore, one of the explanations of LHBC1 (HORVU5Hr1G087250) downregulation might be the link with WRKY33. Another gene identified in our study encodes NAC transcription factor (HORVU4Hr1G051290, FC = 10.72; (Table S3)) potentially targeting *psaA* subunit of PSI (HORVU6Hr1G037020, FC = −5.9; (Table S3)). The up-regulation of genes encoding NAC TFs by oxidative stress was demonstrated [55]. Taking into account that oxidative stress is frequently the secondary effect of severe drought, it can be assumed that in our conditions, the up-regulation of *NAC* leads to negative regulation of the *psaA* expression.

Although all these designated regulatory pairs should be further verified in a wet lab, we pointed here, for the first time in barley, several potential interactions important for our understanding of photosynthesis regulation under drought stress.

Our findings suggested that rapid dehydration treatment induced similar changes in barley photosynthetic machinery as those observed during the onset of drought applied to plants grown in the soil. It opened a new avenue of robust testing of barley genotypes in regard to their sensitivity to water deficit shock in terms of photosynthetic apparatus. We noticed that significant changes concern mainly quantum efficiency of primary photochemistry related to electron trapping (φP_0_), performance index (PI_ABS_), as well as electron transport chain expressed by both quantum efficiencies (φE_0_, ΨE_0_, φR_0,_ and δR_0_) and the value of electron transport flux per cross-section (ET/CS) (Figure 9). Moreover, significant downregulation of genes encoding core-components of photosynthetic machinery such as LHCB1 (HORVU1HR1G088900), LHCA4 (HORVU5HR1G066280), PsaO (HORVU2HR1G073370), and PsbO (HORVU2HR1G057700), together with PsbO (HORVU2HR1G057700), PsbQ (HORVU6HR1G051650), was noted in both our transcriptomic data from microarrays at the onset of drought stress and also after rapid dehydration when selected genes were analyzed using qPCR. Taking these results together. it can be recommended to track early response to drought in barley with the use of rapid dehydration assay instead of soil-applied drought stress.

In our study, exogenously abscisic acid (ABA) profoundly alters OJIP curves in all genotypes tested mainly in the O-J phase of the transient with a well-designated ΔK-band. Therefore, we concluded that the main aspect of photosynthesis affected by ABA is the electron fate between photosynthetic antenna absorption and Q_A_ at the acceptor side of PSII. ABA signal perception and transduction are closely associated with redox signaling and other signaling pathways, and hence, it can be the reason for electron transport disruption [56]. The in vivo study of the effect of ABA on photosynthetic oxygen evolution in barley leaves revealed that ABA influenced the functioning of PSII reaction centers by disrupting chloroplast grana [57]. On the other hand, it was showed that the pretreatment of seedlings with ABA led to an increase in the accumulation of chlorophyll and carotenoid, and it also maintains optimal efficiency of the PSII complex in plants subjected to water stress [58].

One can ask whether the effect observed in our study is in contradiction with the reported protective role of ABA in terms of photosynthesis. The answer seems to be related to the ABA dose since the response to ABA is highly dependent on the dose applied in terms of inhibition or stimulation of, e.g., growth [59]. The 200 µM applied in our study is rather a high concentration that allowed us to observe inhibition of growth by 60% in young barley seedling [30]. However, the application of such a high ABA concentration gave an interesting overview of changes in the photosynthetic apparatus. The effect of a lower ABA concentration applied, 10 µM, did not cause any harmful effect in our study. Therefore, it will be interesting to test it further as a pre-treatment in barley before drought exposure in order to protect the photosynthesis and possibly attenuate the negative effect of stress.

## 4. Materials and Methods

### 4.1. Plant Material

The plant material used in this study included plants of the spring barley cvs. “Sebastian”, “Maresi”, and “Cam/B1//CI008887/CI05761” (designated further as ‘Cam/B1’) analyzed at the seedling stage. The “Sebastian” and “Maresi” are two-rowed, spring barley cultivars developed in Denmark and Germany, respectively. The “Cam/B1” is a two-rowed cultivar derived from Syria (ICARDA, International Centre for Agricultural Research in Dry Areas, Aleppo) that was courteously provided by Prof. Górny (Institute of Plant Genetics, Poznan, Poland). The cvs. “Cam/B1” and “Maresi” are the parents of the mapping population used in our previous studies on mapping the Quantitative Trait Loci (QTLs) that are related to drought-stress tolerance in barley [24] and the cultivar “Sebastian” is the parent of the Targeting Induced Local Lesions in Genomes (TILLING) population (*Hor*TILLUS) developed after chemical mutagenesis at the University of Silesia in Katowice [60]. This population has been used to develop mutants in the genes of interest, including the genes related to abiotic stresses, including drought [22,23].

### 4.2. Assays of Seedling Development in the Presence of Abiotic Stresses

#### 4.2.1. Drought Stress Experiment

Drought stress was applied, as described earlier [23]. Briefly, the experiment was carried out in boxes (400 × 140 × 175 mm) filled with soil containing a mixture of sandy loam and sand (7:2) with known physicochemical properties. The soil was supplied with a nutrient medium. Based on the water retention curve elaborated in the earlier work, the water was easily available for plants at 14% of volumetric water content (vwc) in the soil, whereas the water deficit stress was achieved at 1.5%. When plants were grown in 14% vwc, the relative water content in leaves was approximately 100%. The soil moisture was measured every day using a Time-domain reflectometer (TDR) EasyTest (Institute of Agrophysics, Polish Academy of Sciences, Poland). Plants were grown in a greenhouse for 10 days after sowing (DAS) under optimal water conditions (14% volumetric water content—vwc), 20/18 °C day/night, with a 16/8 h photoperiod and 420 μmol m^−2^ s^−1^ PAR (Photosynthetically active radiation) which was provided by fluorescent lamps. Afterward, the soil moisture was decreased by withholding irrigation under the control of TDR measurements. On 15 DAS (further in the text referred as the onset of drought stress), when the soil moisture decreased to 3%, the plants were moved into a growth chamber, where the temperature regime was set to 25 °C /20 °C day/night, with a 16/8 h photoperiod and 420 μEm^−2^s^−1^ light intensity. The severe drought stress (1.5% vwc) lasted 10 days (16–25 DAS; further in the text referred to as the prolonged drought stress). The control plants were grown under the same conditions with optimal water supply (14% vwc) in parallel to the drought-treated plants. The second leaf was collected from “Sebastian” plants before water withdrawal (10 DAS), at the onset of drought stress (15 DAS) and after severe/prolonged drought (25 DAS), and used for RNA extraction. The physiological analyses were performed at the same time-points. We conducted all analyses on the second leaf since it was already present when plants entered drought treatment. Each genotype was tested in three biological replicates. One box containing 15 plants per genotype was considered as one replicate.

#### 4.2.2. ABA Treatment

The experiment was performed according to the previously described work protocol [30]. Prior to the experiment, the seeds were surface-sterilized with 1% sodium hypochlorite for 20 minutes and then rinsed in water three times for 5 minutes. The experiment was conducted in a growth chamber under the controlled conditions: temperature 22 °C, a 16-h-light/8-h-dark cycle, 200 µmol m^−2^ s^−1^ PAR. A single, sterilized seed was placed in a glass laboratory tube (17 cm length) with a small piece of water-soaked cotton wool at the top and was covered with another glass tube, both of which were wrapped together with parafilm in order to create aeroponic conditions for the developing seedlings. Seedlings were kept at 22 °C with a 16-h-light/8-h-dark cycle for four days. Then, four-day-old seedlings were transferred into new laboratory glass tubes containing 30 mL of a liquid MS (Murachige and Skoog) medium with or without ABA (10 and 200 µM). After two days, the solutions were replaced with fresh ones. The chlorophyll *a* fluorescence was measured on the 5th day after transfer. The experiment was conducted three times. The seeds that were used for the experiments had been harvested and stored for the same time period. The average number of seedlings that were analyzed in one replicate was six per concentration.

#### 4.2.3. Rapid Dehydration Assay

The experiment was conducted in a growth chamber under the controlled conditions: 25 °C/20 °C day/night, with a 16/8 h photoperiod and 420 μmol m^−2^s^−1^ PAR. A second leaf was detached from two-week-old barley seedling and weighed in order to obtain fresh weight (Fw). Next, it was dehydrated by placing immediately under the airflow at 25 °C. The dehydration was conducted until the 30% loss of weight according to earlier elaborated protocol. After approximately four hours, the leaf was weighed again in order to obtain the dry weight (Dw). The percentage of water loss was calculated according to the formula: WL = Dw*100%/Fw. The experiment was performed in three biological replicates for each genotype tested. Each biological replicate consisted of three leaves. Next, the chlorophyll *a* fluorescence was measured (according to the protocol described below) in both detached and control leaves, and then the tissue was frozen in liquid nitrogen for further RNA extraction and gene expression analyses with the use of the qPCR technique.

### 4.3. Transcriptomic Analyses

#### 4.3.1. Microarray Experiment and Bioinformatics Analysis

RNA was extracted from the second leaf (50–100 mg) of “Sebastian” plants collected on 10, 15, and 25 DAS in three biological replications (each biological replicate represented leaf of one seedling). RNA isolation was conducted using the TriPure reagent, according to the modified Chomczynski’s method [61]. For the microarray analyses, RNA was additionally purified using the precipitation in 1 M lithium chloride, and each RNA precipitate was then dissolved in 15 μL of nuclease-free H_2_O. For RNAseq analyses, RNA was extracted using miRvana isolation kit (ThermoFisher Scientific, Waltham, Massachusetts, USA) according to the manufacturer’s protocol. A NanoDrop (ND-1000) spectrophotometer (NanoDrop Technologies, Wilmington, USA) was used for concentration quantification and quality check. The RNA integrity was analyzed using Agilent 2100 Bioanalyzer with RNA 6000 Nanochip (Agilent Technologies, Santa Clara, CA, USA).

The synthesis, labeling, and hybridization of cDNA and cRNA were carried out at the Genomics Core Facility, European Molecular Biology Laboratory (EMBL), Heidelberg, Germany. Barley Gene Expression Microarray, 4x44K from Agilent Technologies, was employed. As described in [62], 18,000 array probes were mapped to cDNAs that represented the 11,340 unique high-confidence (HC) genes that have been annotated in the barley genome. Taking into account that the estimated number of HC barley genes is 39 734 [26], the Agilent Barley Gene Expression Microarray represents about 30% of barley genes. The microarray data were analyzed using GeneSpring GX 13.0 software (Agilent Technologies, Santa Clara, California, United States). Hybridization data from all of the biological replicates were subjected to a per chip normalization using the percentile shift method to the 75th percentile. A baseline transformation was then performed on the median of all of the samples. The annotation of the Agilent Barley Gene Expression Microarray (Agilent Technologies, Santa Clara, California, United States) was performed against the Ensembl Plants database and IBSC v2. Barley genome.

Differentially expressed transcripts were identified using the R/Bioconductor package “edgeR” (v. 3.3.3; [63]) with Benjamini–Hochberg false discovery rate (FDR) ≤ 0.01. The absolute log_2_-fold change > 2 was required to define a gene as differentially expressed (DEG) among the analyzed conditions (onset of drought stress (15 DAS) vs. control (10 DAS); severe drought stress (25 DAS) vs. control (10 DAS)). Hierarchical clustering and a volcano plot were generated using the R/Bioconductor package “ggplots” (version 3.3.3; https://cran.r-project.org/web/packages/gplots/gplots.pdf). K-Means clustering of the top 1000 ranked genes (based on standard deviation) followed by an enrichment analysis was performed using the shiny app iDEP8.0 (http://bioinformatics.sdstate.edu/idep).

#### 4.3.2. RNAseq Experiment and Bioinformatics Data Analysis

RNA was extracted from the second leaf (50–100 mg) of “Sebastian” plants collected on 10 and 25 DAS in three biological replications (each biological replicate represented leaf of one seedling). RNA isolation was conducted using the MirVana isolation kit (ThermoFisher Scientific) according to the manufacturer protocol. NanoDrop (ND-1000) spectrophotometer (NanoDrop Technologies, Wilmington, USA) was used for concentration quantification and quality check. The RNA integrity was analyzed using Agilent 2100 Bioanalyzer with RNA 6000 Nanochip (Agilent Technologies, Santa Clara, USA).

cDNA libraries were prepared following Illumina TruSeq standard procedures and sequenced in an Illumina HiSeq4000 sequencer at the Centre for Bioinformatics and Data Analysis, the Medical University of Bialystok, using a full flow-cell, six samples per lane, to produce 2 × 100 bp paired-end reads. The whole dataset consisted of three biological replicates for each condition (control and drought).

Raw reads were processed with the Fast QC software2 (v0.11.5, Cambridge, UK), and then trimmed by removing all adaptor sequences, empty reads, and low quality reads (Q < 30 and length < 50 bp) to obtain clean reads with the use of the CLC Genomics Workbench software (v. 5.0 Qiagen, Vedbæk, Denmark). The clean reads were mapped and quantified against the Barley reference transcriptome using Kallisto v 0.43.0 [64] in default mode with 100 times bootstrapping. Analysis of differential gene expression was carried out with DESeq2 [65]. Differentially expressed genes (DEGs) were required to have log_2_ fold change ≥ 2 or ≤ –2 between contrasted conditions and an adjusted *p*-value ≤ 0.01 after Benjamini–Hochberg correction. Enrichment of gene ontology-based on GO terms was performed using the Singular Enrichment Analysis (SEA) available through the AgriGO2 toolkit [66] (http://systemsbiology.cau.edu.cn/agriGOv2/). The analysis was performed using the Fisher test, the Multi-test adjustment method Yekutelli (FDR under dependency) at a significance level of p ≤ 0.05, and minimum mapping entries of five.

PlantTFcat (https://plantgrn.noble.org/PlantTFcat/familylist.do) was used to identify transcription factors (TFs) within the lists of up- and down-regulated genes [67]. Next, the PlantRegMap together with FIMO (Find Individual Motif Occurrences) tool (http://plantregmap.cbi.pku.edu.cn/binding_site_prediction.php), were employed in order to search for TF binding Motifs (derived from experiments that are projected to 156 species using BLAST_reciprocal best hits) in promoter sequences of photosynthesis-related genes. These sequences were retrieved using BioMart tool implemented in EnsemblPlants v.43 using R studio. FIMO program computes a log-likelihood ratio score for each motif with respect to each sequence position and converts these scores to *p*-values using dynamic programming. Finally, FIMO employs a bootstrap method to estimate false discovery rates (FDRs). Because the FDR is not monotonic relative to the *p*-value, FIMO instead reports for each *p*-value, a corresponding q-value, which is defined as the minimal FDR threshold at which the *p*-value is deemed significant. The obtained results were then cross-checked with the TFs list that was identified within the list of DEGs in order to retrieve the TFs (within DEGs) that are able to bind promoter regions of photosynthesis-related genes identified in our study.

#### 4.3.3. Quantitative Real-Time PCR Analysis

The analysis of expression of photosynthesis-related genes was performed in two-week-old barley seedlings of three cultivars “Sebastian”,”Cam/B1”, and “Maresi” exposed to rapid dehydration assay. One microgram of total RNA was used in 20 μl reactions for cDNA synthesis using a Maxima First Strand cDNA synthesis Kit for RT-qPCR (Thermo Scientific; Waltham, Massachusetts, United States). The cDNA was diluted 1:5 with ddH_2_O and used as the template for the quantitative PCR. All of the primers used in the qPCR were designed using Primer3 software (http://bioinfo.ut.ee/primer3-0.4.0/). The 10 μL qPCR reaction mix contained 2 μl of diluted cDNA, 1 μl of the primer pair mixture (5 μM) and 5 μL of 2 × Master Mix (LightCycler 480 SYBR Green I Master; Roche). The following qPCR protocol was used on a LightCycler 480 Real-Time PCR Instrument (Roche) using the SYBR Green I method: initial denaturation for 10 min at 95 °C, followed by 45 cycles of 10 s at 95 °C, 15 s at 60 °C, and 10 s at 72 °C, followed by a melting-curve analysis. The reference gene that was used in this study was *EF1* (Elongation factor 1-a; HORVU6Hr1G085370; [68]). Data were analyzed using LinRegPCR [69] and Excel software (Microsoft Office). Calculations of the fold change of expression (FC) were done using the formula FC = E-^ΔCt^, where E is the mean value of the amplification efficiency of a given gene and ΔCt corresponds to the difference between the mean Ct-values of all of the biological replicates between the two samples that were compared, precisely dehydrated vs. control.

### 4.4. Chlorophyll a Fluorescence and Chlorophyll Content Measurements

Chlorophyll *a* fluorescence was measured using a Plant Efficiency Analyser (PocketPEA fluorimeter, Hansatech Instruments Ltd., England). Measurements were taken in three biological replicates; each consisted of the second leaf of three plants. Chlorophyll *a* fluorescence was measured in drought-, dehydration-, and ABA-treated plants at the indicated time-points. Before the measurements, the leaves were dark-adapted for 30 min and then were exposed to a pulse of saturating light at an intensity of 3500 μmol m^−2^ s^−1^ with a peak wavelength of 627 nm. Further analysis of chlorophyll *a* fluorescence signal was based on the JIP-test concept. The primary photochemistry of PSII was further evaluated using the parameters described in Table 2 [18]. The changes in these parameters have been widely demonstrated to be associated with various stressors action and plant vitality [15,20,21,24,69,70,71]. The OJIP curves were plotted as relative variable fluorescence, ΔV_t_ where V_t_ = (F_t_ − F_0_)/(F_M_ − F_0_) according to [70] and ΔV_t_ was calculated as the difference in the variable fluorescence that was obtained by subtracting the fluorescence values recorded in control and stressed conditions [71], for abbreviations see Table 2). The polyphenol and chlorophyll meter Dualex Scientific+^TM^ (Force-A, France) was used to measure chlorophyll content in leaves. The stomatal conductance (mmol m^−2^ s^−1^) of the leaves was determined using an AP4 porometer (Delta-T Devices, Burwell, UK) before midday (referred to the photoperiod in the growth chamber). The measurements were performed in the center of fully expanded second leaves. For each genotype studied, nine leaves (three per replication) were measured on the adaxial side.

### 4.5. Statistical Analysis

Statistical analyses were performed using ANOVA (*p* < 0.05) followed by Tukey’s honestly significant difference test (Tukey HSD test) (*p* ≤ 0.05) to assess the differences between the analyzed groups of values. All of the statistical analyses were performed using the STATISTICA software, version 13.1 (Dell USA, 2016, www.statsoft.com).

## 5. Conclusions

Although the negative impact of drought on photosynthesis process is known, our work delivered comprehensive information related to photosynthesis process along with detailed genetic dissection of barley under drought stress

Remarkably, for the first time, we identified and annotated almost 150 barley genes encoding crucial components of photosystems, electron transfer chain, together with Calvin cycle enzymes. Moreover, we designated putative 17 regulatory pairs between transcription factors identified within DEG sets with discovered TFBS within promoter sequences in photosynthesis-related genes found in our study. These results together can be considered as a significant resource for further studies concerning photosynthetic efficiency in barley and other crops under environmental stresses.

Taking advantage of the combined physiological and transcriptomics analyses, we were able to integrate the data and link gene expression level with changes of the measured photosynthetic parameters. Moreover, we also verified our results using genetically distant barley cultivars, and we obtained results pointing to the conserved mechanism of the photosynthesis process under drought stress.

## Figures and Tables

**Table 1:** Putative regulatory pairs between Differentially Expressed Genes (DEGs) encoding transcription factors and predicted targets identified within photosynthesis-related genes.

Transcription Factor Gene ID	FC of TF Gene	FDR	TF Family	Description of TF Gene	Target Gene ID	Target Description	FC of Target Gene	FDR
HORVU4Hr1G078410	−20.36	2.25 × 10^−15^	HD-ZIP	Homeobox-leucine zipper protein family	HORVU2Hr1G010630	ribulose-bisphosphate carboxylase small chain [EC:4.1.1.39]	−432.2	1.56 × 10^−4^
HORVU1Hr1G073300	−12.71	7.62 × 10^−5^	MYB_related	myb-like transcription factor family protein	HORVU1Hr1G035720	ribulose-bisphosphate carboxylase small chain [EC:4.1.1.39]	−37.5	1.26 × 10^−84^
HORVU6Hr1G066000	−8.18	9.07 × 10^−5^	MYB_related	myb-like transcription factor family protein	HORVU1Hr1G067300	phosphoglycerate kinase [EC:2.7.2.3]	−52.7	2.27 × 10^−32^
HORVU6Hr1G019650	−7.20	8.16 × 10^−6^	G2-like	myb-like transcription factor family protein	HORVU5Hr1G109260	light-harvesting complex II chlorophyll a/b binding protein 1	−1357	5.10 × 10^−37^
HORVU5Hr1G014170	−5.41	5.13 × 10^−4^	bZIP	Basic-leucine zipper (bZIP) transcription factor family protein	HORVU2Hr1G060480	photosystem I subunit X	−54.7	2.56 × 10^−27^
HORVU3Hr1G088200	−3.78	3.03 × 10^−3^	WRKY	WRKY DNA-binding protein 33	HORVU5Hr1G087250	light-harvesting complex II chlorophyll a/b binding protein 1	−13.1	1.86 × 10^−6^
HORVU3Hr1G032440	−3.26	8.28 × 10^−4^	G2-like	Two-component response regulator-like APRR2	HORVU2Hr1G075200	F-type H+-transporting ATPase subunit b	−52.7	2.84 × 10^−16^
HORVU2Hr1G060680	−2.62	6.33 × 10^−4^	bHLH	Transcription factor PIF5	HORVU6Hr1G033160	light-harvesting complex I chlorophyll a/b binding protein 3	−30.2	5.16 × 10^−11^
HORVU6Hr1G074970	−2.37	2.93 × 10^−3^	ERF	Ethylene-responsive transcription factor 1	HORVU3Hr1G013350	triosephosphate isomerase (TIM) [EC:5.3.1.1]	−6.7	8.10 × 10^−16^
HORVU4Hr1G052330	2.46	3.42 × 10^−3^	bZIP	transcription factor-related	HORVU2Hr1G063740	glutamate--glyoxylate aminotransferase [EC:2.6.1.4 2.6.1.2 2.6.1.44]	−8.0	2.03 × 10^−11^
HORVU1Hr1G074960	2.80	4.37 × 10^−8^	bZIP	G-box binding factor 4	HORVU1Hr1G021830	cytochrome b6-f complex subunit 4	−4.3	1.49 × 10^−3^
HORVU5Hr1G036330	3.05	7.45 × 10^−4^	GATA	GATA transcription factor 9	HORVU6Hr1G051650	photosystem II oxygen-evolving enhancer protein 3	−202.0	7.00 × 10^−4^
HORVU1Hr1G090030	3.06	8.67 × 10^−8^	bZIP	G-box binding factor 2	HORVU2Hr1G062090	cytochrome b6	−6.7	6.65 × 10^−9^
HORVU4Hr1G020540	3.12	2.20 × 10^−4^	bZIP	Transcription factor VIP1	HORVU2Hr1G043240	photosystem II P680 reaction center D1 protein [EC:1.10.3.9]	−5.9	5.99 × 10^−13^
HORVU2Hr1G119610	3.53	4.62 × 10^−4^	MYB_related	myb-like transcription factor family protein	HORVU2Hr1G043240	photosystem II P680 reaction center D1 protein [EC:1.10.3.9]	−5.9	5.99 × 10^−13^
HORVU4Hr1G051290	10.72	1.46 × 10^−9^	NAC	NAC domain protein.	HORVU6Hr1G037020	photosystem I P700 chlorophyll a apoprotein A1	−5.9	4.15 × 10^−7^
HORVU2Hr1G021080	13.16	5.84 × 10^−7^	bZIP	Basic-leucine zipper (bZIP) transcription factor family protein	HORVU2Hr1G062090	cytochrome b6	−6.7	6.65 × 10^−9^

**Table 2:** Definition of the terms and formulas for calculation of the JIP-test parameters from the chlorophyll *a* fluorescence transient OJIP emitted by dark-adapted leaves.

**Quantum Yields and Probabilities**	
ϕP_0_ = 1 − F_0_/F_M_	Maximum quantum yield of primary photochemistry in PSII (at t = 0)
ϕE_0_ = (1 − F_0_/F_M_)(1 − V_J_)	Quantum yield of electron transport (at t = 0) beyond Q_A_^−^
ϕR_0_ = (1 − F_0_/F_M_)(1 − V_I_)	Quantum yield for reduction of the end of electron acceptors at PSI acceptor side (RE)
ϕD_0_ = F_0_/F_M_	Quantum yield (at t = 0) of energy dissipation
δR_0_ = (1 − V_I_)/(1 − V_J_)	The efficiency with which an electron can move from the reduced intersystem electron acceptors to the PSI end electron acceptors
ψE_0_ = 1 − V_J_	Probability (at t = 0) that a trapped exciton will move an electron into electron transport chain beyond Q_A_^−^
**Specific Energy fluxes expressed per active PSII reaction center (RC)**	
ABS/RC = (1 − γ_RC_)/ γ_RC_ = M_0_ × (1/V_J_)/[1 − (F_0_/F_M_)]	Apparent antenna size of active PSII RC
TR_0_/RC = M_o_(1/V_j_)	Trapping flux (leading to Q_A_ reduction) per RC
DI_0_/RC = (ABS/RC − TR_0_/RC)	Dissipated energy flux per RC
ET_0_/RC = M_0_(1/V_j_) ψ_0_	Electron transport flux per RC (further than Q_A_^−^)
RC/CS_0_ = F_O_ φ_P0_ V_j_/_M0_	Density of RCs (Q_A_^−^ reducing PSII reaction centers)
**Performance index per absorption**	
PI_ABS_ = 1 − (F_0_/F_M_)/M_o_/V_J_ × F_M_ − F_0_/F_0_ × 1 − V_J_/V_J_	Performance index (potential) for energy conservation from exciton to the reduction of intersystem electron acceptors
**Phenomenological energy fluxes per excited cross-section (CS)**	
ABS/CS	Absorption energy flux per CS
TR_0_/CS	Trapped energy flux per CS
ET_0_/CS	Electron transport flux per CS
DI_0_/CS	Dissipation energy flux per CS

F_0_—fluorescence intensity at 50 µs, F_M_—maximal fluorescence intensity, F_J_—fluorescence intentisity at J step (at 2 ms), V_J_—relative variable fluorescence at 2 ms calculated as V_J_ = (F_J_ − F_0_)/(F_M_ − F_o_), M_o_ initial slope of fluorescence kinetics which can be derived from equatation M_o_ = 4 × (F300µs − F_0_)/(F_M_ − F_0_).

## Supplementary Materials

Supplementary materials can be found at https://www.mdpi.com/1422-0067/20/24/6341/s1.
