# Supplementary material for: Genetic and Physiological Dissection of Photosynthesis in Barley Exposed to Drought Stress
Source: Int J Mol Sci. 2019 Dec 16;20(24):6341. doi: 10.3390/ijms20246341 (PMC6940956; doi:10.3390/ijms20246341)
Supplement: Supplementary file 1 [file ijms-20-06341-s001.zip › ADG_photosynthesis_TableS1.docx]

| **Table S2. DEGs identified using RNA-seq as photosynthesis related downregulated under drought in barley** | | | | | | | | |
| --- | --- | --- | --- | --- | --- | --- | --- | --- |
| **Components** | **Ensembl ID** | **KO KEGG** | **name** | **Description** | **log2 Fold Change** | **Fold Change** | **Adj.Pval** | **Encoded by N/C genome** |
| **Calvin cycle** | HORVU4Hr1G060630 | K03841 | FBP | fructose-1,6-bisphosphatase I [EC:3.1.3.11] | -3,4 | -10,3 | 9,62E-14 | N |
|  | HORVU4Hr1G019570 | K01623 | ALDO | fructose-bisphosphate aldolase, class I [EC:4.1.2.13] | -3,6 | -11,8 | 9,13E-14 | N |
|  | HORVU5Hr1G032980 | K01623 | ALDO | fructose-bisphosphate aldolase, class I [EC:4.1.2.13] | -4,2 | -18,2 | 1,29E-11 | N |
|  | HORVU2Hr1G063740 | K14272 | GGAT | glutamate--glyoxylate aminotransferase [EC:2.6.1.4 2.6.1.2 2.6.1.44] | -3,0 | -8,0 | 2,03E-11 | N |
|  | HORVU6Hr1G032070 | K00134 | GAPDH | glyceraldehyde 3-phosphate dehydrogenase [EC:1.2.1.12] | -2,3 | -5,1 | 1,05E-15 | N |
|  | HORVU4Hr1G082700 | K05298 | GAPA | glyceraldehyde-3-phosphate dehydrogenase (NADP+) (phosphorylating) [EC:1.2.1.13] | -4,5 | -22,9 | 1,3E-20 | N |
|  | HORVU1Hr1G045720 | K00028 | E1.1.1.39 | malate dehydrogenase (decarboxylating) [EC:1.1.1.39] | -2,1 | -4,3 | 1,31E-09 | N |
|  | HORVU3Hr1G019790 | K00029 | E1.1.1.40 | malate dehydrogenase (oxaloacetate-decarboxylating)(NADP+) [EC:1.1.1.40] | -2,6 | -6,2 | 9,31E-19 | N |
|  | HORVU1Hr1G081550 | K00026 | MDH2 | malate dehydrogenase [EC:1.1.1.37] | -3,4 | -10,5 | 4,87E-15 | N |
|  | HORVU1Hr1G090710 | K00026 | MDH2 | malate dehydrogenase [EC:1.1.1.37] | -2,7 | -6,7 | 1,35E-13 | N |
|  | HORVU5Hr1G098890 | K00026 | MDH2 | malate dehydrogenase [EC:1.1.1.37] | -2,7 | -6,4 | 2,09E-08 | N |
|  | HORVU4Hr1G062440 | K01610 | E4.1.1.49 | phosphoenolpyruvate carboxykinase (ATP) [EC:4.1.1.49] | -3,2 | -9,0 | 1,96E-12 | N |
|  | HORVU3Hr1G029200 | K01595 | ppc | phosphoenolpyruvate carboxylase [EC:4.1.1.31] | -3,2 | -8,9 | 4,93E-07 | N |
|  | HORVU1Hr1G067300 | K00927 | PGK | phosphoglycerate kinase [EC:2.7.2.3] | -5,7 | -52,7 | 2,27E-32 | N |
|  | HORVU6Hr1G067660 | K00855 | PRK | phosphoribulokinase [EC:2.7.1.19] | -4,7 | -25,1 | 4,81E-17 | C |
|  | HORVU2Hr1G066480 | K01807 | rpiA | ribose 5-phosphate isomerase A [EC:5.3.1.6] | -3,2 | -9,4 | 8,6E-18 | C |
|  | HORVU2Hr1G084620 | K01601 | rbcL | ribulose-bisphosphate carboxylase large chain [EC:4.1.1.39] | -5,9 | -58,7 | 1,39E-38 | C |
|  | HORVU2Hr1G084630 | K01601 | rbcL | ribulose-bisphosphate carboxylase large chain [EC:4.1.1.39] | -2,5 | -5,5 | 3,94E-06 | C |
|  | HORVU3Hr1G048820 | K01601 | rbcL | ribulose-bisphosphate carboxylase large chain [EC:4.1.1.39] | -6,3 | -80,5 | 1,79E-25 | C |
|  | HORVU3Hr1G055820 | K01601 | rbcL | ribulose-bisphosphate carboxylase large chain [EC:4.1.1.39] | -3,7 | -13,1 | 3,1E-13 | C |
|  | HORVU5Hr1G064700 | K01601 | rbcL | ribulose-bisphosphate carboxylase large chain [EC:4.1.1.39] | -5,6 | -50,2 | 2,27E-18 | C |
|  | HORVU6Hr1G047870 | K01601 | rbcL | ribulose-bisphosphate carboxylase large chain [EC:4.1.1.39] | -6,0 | -66,0 | 5,09E-21 | C |
|  | HORVU6Hr1G049260 | K01601 | rbcL | ribulose-bisphosphate carboxylase large chain [EC:4.1.1.39] | -5,4 | -41,5 | 1,43E-20 | C |
|  | HORVU1Hr1G035720 | K01602 | rbcS | ribulose-bisphosphate carboxylase small chain [EC:4.1.1.39] | -5,2 | -37,5 | 1,26E-84 | N |
|  | HORVU2Hr1G010630 | K01602 | rbcS | ribulose-bisphosphate carboxylase small chain [EC:4.1.1.39] | -8,8 | -432,2 | 0,000156 | N |
|  | HORVU2Hr1G010670 | K01602 | rbcS | ribulose-bisphosphate carboxylase small chain [EC:4.1.1.39] | -9,9 | -941,3 | 1,54E-86 | N |
|  | HORVU2Hr1G010690 | K01602 | rbcS | ribulose-bisphosphate carboxylase small chain [EC:4.1.1.39] | -8,7 | -414,0 | 3,1E-112 | N |
|  | HORVU5Hr1G050990 | K01602 | rbcS | ribulose-bisphosphate carboxylase small chain [EC:4.1.1.39] | -6,3 | -80,7 | 1,16E-55 | N |
|  | HORVU5Hr1G051010 | K01602 | rbcS | ribulose-bisphosphate carboxylase small chain [EC:4.1.1.39] | -6,5 | -88,3 | 5,75E-49 | N |
|  | HORVU4Hr1G074810 | K01783 | rpe | ribulose-phosphate 3-epimerase [EC:5.1.3.1] | -3,4 | -10,6 | 2,36E-21 |  |
|  | HORVU3Hr1G085270 | K01100 | E3.1.3.37 | sedoheptulose-bisphosphatase [EC:3.1.3.37] | -5,0 | -31,6 | 3,42E-17 | C |
|  | HORVU3Hr1G013350 | K01803 | TPI | triosephosphate isomerase (TIM) [EC:5.3.1.1] | -2,8 | -6,7 | 8,1E-16 | C |
|  | HORVU5Hr1G082630 | K01803 | TPI | triosephosphate isomerase (TIM) [EC:5.3.1.1] | -2,5 | -5,8 | 4,82E-08 | C |
| **cytochrome b6f complex** | HORVU2Hr1G004590 | K02634 | petA | apocytochrome f | -4,6 | -24,3 | 1,7E-09 | C |
|  | HORVU5Hr1G065140 | K02634 | petA | apocytochrome f | -3,4 | -10,4 | 2,48E-17 | C |
|  | HORVU6Hr1G049300 | K02634 | petA | apocytochrome f | -3,5 | -11,3 | 1,77E-15 | C |
|  | HORVU1Hr1G003510 | K02635 | petB | cytochrome b6 | -2,9 | -7,6 | 2,73E-06 | C |
|  | HORVU1Hr1G063040 | K02635 | petB | cytochrome b6 | -4,4 | -20,7 | 8,02E-11 | C |
|  | HORVU2Hr1G062090 | K02635 | petB | cytochrome b6 | -2,8 | -6,7 | 6,65E-09 | C |
|  | HORVU5Hr1G065020 | K02635 | petB | cytochrome b6 | -2,6 | -5,9 | 2,73E-07 | C |
|  | HORVU6Hr1G049420 | K02635 | petB | cytochrome b6 | -2,8 | -7,1 | 8,53E-08 | C |
|  | HORVU2Hr1G041610 | K02636 | PetC | cytochrome b6-f complex iron-sulfur subunit [EC:7.1.1.6] | -3,3 | -9,9 | 9,48E-16 | N |
|  | HORVU1Hr1G021830 | K02637 | petD | cytochrome b6-f complex subunit 4 | -2,1 | -4,3 | 0,00149 | C |
|  | HORVU2Hr1G039510 | K08906 | petJ | cytochrome c6 | -2,3 | -4,9 | 0,00053 | C |
|  | HORVU2Hr1G072880 | K02639 | petF | ferredoxin | -6,9 | -122,3 | 4,92E-13 | N |
|  | HORVU4Hr1G004210 | K02639 | petF | ferredoxin | -2,3 | -4,9 | 3,67E-06 | N |
|  | HORVU5Hr1G103180 | K02641 | petH | ferredoxin--NADP+ reductase [EC:1.18.1.2] | -1,9 | -3,7 | 3,51E-05 | N |
|  | HORVU6Hr1G000620 | K02641 | petH | ferredoxin--NADP+ reductase [EC:1.18.1.2] | -4,4 | -21,6 | 3,62E-15 | N |
|  | HORVU7Hr1G002210 | K02641 | petH | ferredoxin--NADP+ reductase [EC:1.18.1.2] | -5,1 | -34,7 | 7,01E-18 | N |
|  | HORVU7Hr1G000900 | K02638 | petE | plastocyanin | -5,3 | -39,8 | 8,9E-15 | N |
| **ATP synthase** | HORVU1Hr1G038040 | K02111 | ATPF1A | F-type H+/Na+-transporting ATPase subunit alpha [EC:7.1.2.2 7.2.2.1] | -4,0 | -15,7 | 4,8E-31 | C |
|  | HORVU4Hr1G045340 | K02111 | ATPF1A | F-type H+/Na+-transporting ATPase subunit alpha [EC:7.1.2.2 7.2.2.1] | -4,5 | -22,1 | 3,87E-40 | C |
|  | HORVU5Hr1G017350 | K02111 | ATPF1A | F-type H+/Na+-transporting ATPase subunit alpha [EC:7.1.2.2 7.2.2.1] | -6,5 | -91,4 | 1,39E-15 | C |
|  | HORVU6Hr1G049170 | K02111 | ATPF1A | F-type H+/Na+-transporting ATPase subunit alpha [EC:7.1.2.2 7.2.2.1] | -5,1 | -34,5 | 7,79E-33 | C |
|  | HORVU3Hr1G053770 | K02112 | ATPF1B | F-type H+/Na+-transporting ATPase subunit beta [EC:7.1.2.2 7.2.2.1] | -4,5 | -22,0 | 3,56E-06 | C |
|  | HORVU4Hr1G016830 | K02112 | ATPF1B | F-type H+/Na+-transporting ATPase subunit beta [EC:7.1.2.2 7.2.2.1] | -4,9 | -30,1 | 8,05E-11 | C |
|  | HORVU5Hr1G064690 | K02112 | ATPF1B | F-type H+/Na+-transporting ATPase subunit beta [EC:7.1.2.2 7.2.2.1] | -5,9 | -59,0 | 3,72E-13 | C |
|  | HORVU6Hr1G047880 | K02112 | ATPF1B | F-type H+/Na+-transporting ATPase subunit beta [EC:7.1.2.2 7.2.2.1] | -2,3 | -4,8 | 0,00032 | C |
|  | HORVU4Hr1G045380 | K02108 | ATPF0A | F-type H+-transporting ATPase subunit a | -2,9 | -7,3 | 3,08E-05 | C |
|  | HORVU5Hr1G067560 | K02108 | ATPF0A | F-type H+-transporting ATPase subunit a | -4,6 | -24,2 | 2,25E-10 | C |
|  | HORVU6Hr1G049150 | K02108 | ATPF0A | F-type H+-transporting ATPase subunit a | -4,5 | -22,9 | 3,46E-11 | C |
|  | HORVU1Hr1G003520 | K02109 | ATPF0B | F-type H+-transporting ATPase subunit b | -4,2 | -18,3 | 7,42E-11 | C |
|  | HORVU2Hr1G075200 | K02109 | ATPF0B | F-type H+-transporting ATPase subunit b | -5,7 | -52,7 | 2,84E-16 | C |
|  | HORVU3Hr1G096520 | K02109 | ATPF0B | F-type H+-transporting ATPase subunit b | -2,6 | -6,0 | 3,06E-09 | C |
|  | HORVU4Hr1G058970 | K02109 | ATPF0B | F-type H+-transporting ATPase subunit b | -3,1 | -8,5 | 1,51E-11 | C |
|  | HORVU3Hr1G063590 | K02133 | ATPeF1B | F-type H+-transporting ATPase subunit beta [EC:7.1.2.2] | -1,2 | -2,4 | 0,00206 | C |
|  | HORVU6Hr1G049160 | K02110 | ATPF0C | F-type H+-transporting ATPase subunit c | -4,2 | -18,1 | 1,75E-19 | N |
|  | HORVU2Hr1G072650 | K02138 | ATPeF0D | F-type H+-transporting ATPase subunit d | -1,6 | -3,1 | 6,91E-05 | N |
|  | HORVU3Hr1G053790 | K02114 | ATPF1E | F-type H+-transporting ATPase subunit epsilon | -5,5 | -45,6 | 3,55E-27 | C |
|  | HORVU6Hr1G022990 | K02114 | ATPF1E | F-type H+-transporting ATPase subunit epsilon | -2,3 | -5,1 | 0,00457 | C |
|  | HORVU6Hr1G049250 | K02114 | ATPF1E | F-type H+-transporting ATPase subunit epsilon | -5,4 | -43,5 | 2,01E-17 | C |
|  | HORVU1Hr1G031600 | K02136 | ATPeF1G | F-type H+-transporting ATPase subunit gamma | -1,5 | -2,8 | 0,000416 | N |
| **PSI-LHCI complex** | HORVU1Hr1G038010 | K02689 | psaA | photosystem I P700 chlorophyll a apoprotein A1 | -2,7 | -6,4 | 4,87E-11 | C |
|  | HORVU3Hr1G004680 | K02689 | psaA | photosystem I P700 chlorophyll a apoprotein A1 | -3,0 | -7,8 | 4,74E-21 | C |
|  | HORVU3Hr1G055050 | K02689 | psaA | photosystem I P700 chlorophyll a apoprotein A1 | -2,9 | -7,7 | 7,02E-10 | C |
|  | HORVU4Hr1G045310 | K02689 | psaA | photosystem I P700 chlorophyll a apoprotein A1 | -3,1 | -8,7 | 2,48E-07 | C |
|  | HORVU5Hr1G064640 | K02689 | psaA | photosystem I P700 chlorophyll a apoprotein A1 | -2,9 | -7,6 | 4,56E-18 | C |
|  | HORVU6Hr1G037020 | K02689 | psaA | photosystem I P700 chlorophyll a apoprotein A1 | -2,6 | -5,9 | 4,15E-07 | C |
|  | HORVU6Hr1G041340 | K02689 | psaA | photosystem I P700 chlorophyll a apoprotein A1 | -3,6 | -12,4 | 4,36E-05 | C |
|  | HORVU6Hr1G049200 | K02689 | psaA | photosystem I P700 chlorophyll a apoprotein A1 | -2,9 | -7,6 | 4,31E-29 | C |
|  | HORVU3Hr1G004700 | K02690 | psaB | photosystem I P700 chlorophyll a apoprotein A2 | -3,1 | -8,3 | 4,33E-27 | C |
|  | HORVU4Hr1G045300 | K02690 | psaB | photosystem I P700 chlorophyll a apoprotein A2 | -2,9 | -7,7 | 9,46E-29 | C |
|  | HORVU6Hr1G049190 | K02690 | psaB | photosystem I P700 chlorophyll a apoprotein A2 | -3,2 | -8,9 | 1,16E-17 | C |
|  | HORVU5Hr1G109710 | K02692 | PsaD | photosystem I subunit II | -3,9 | -14,9 | 5,39E-10 | N |
|  | HORVU5Hr1G100140 | K02694 | PsaF | photosystem I subunit III | -4,0 | -16,1 | 7,69E-17 | N |
|  | HORVU2Hr1G019820 | K02701 | PsaN | photosystem I subunit PsaN | -4,5 | -22,8 | 8,08E-16 | N |
|  | HORVU2Hr1G073370 | K14332 | PsaO | photosystem I subunit PsaO | -7,3 | -155,7 | 4,07E-25 | N |
|  | HORVU5Hr1G071920 | K08905 | PsaG | photosystem I subunit V | -3,8 | -14,4 | 9,89E-16 | N |
|  | HORVU2Hr1G044740 | K02691 | psaC | photosystem I subunit VII | -3,1 | -8,8 | 7,77E-14 | C |
|  | HORVU5Hr1G005480 | K02691 | psaC | photosystem I subunit VII | -3,1 | -8,8 | 0,000015 | C |
|  | HORVU2Hr1G060480 | K02698 | PsaK | photosystem I subunit X | -5,8 | -54,7 | 2,56E-27 | N |
|  | HORVU3Hr1G009210 | K02699 | PsaL | photosystem I subunit XI | -4,5 | -22,3 | 4,3E-18 | N |
|  | HORVU2Hr1G036960 | K08908 | LHCA2 | light-harvesting complex I chlorophyll a/b binding protein 2 | -4,5 | -22,4 | 4,16E-16 | N |
|  | HORVU5Hr1G062240 | K08908 | LHCA2 | light-harvesting complex I chlorophyll a/b binding protein 2 | -3,2 | -9,0 | 9,77E-11 | N |
|  | HORVU6Hr1G033160 | K08909 | LHCA3 | light-harvesting complex I chlorophyll a/b binding protein 3 | -4,9 | -30,2 | 5,16E-11 | N |
|  | HORVU5Hr1G066280 | K08910 | LHCA4 | light-harvesting complex I chlorophyll a/b binding protein 4 | -6,2 | -72,9 | 1,07E-10 | N |
| **PSII-LHCII complex** | HORVU6Hr1G085170 | K03541 | PsbR | photosystem II 10kDa protein | -3,7 | -13,1 | 9,31E-19 | N |
|  | HORVU3Hr1G082740 | K03542 | PsbS | photosystem II 22kDa protein | -2,2 | -4,5 | 0,000172 | N |
|  | HORVU2Hr1G062030 | K02705 | psbC | photosystem II CP43 chlorophyll apoprotein | -2,0 | -4,0 | 1,23E-13 | C |
|  | HORVU5Hr1G065050 | K02704 | psbB | photosystem II CP47 chlorophyll apoprotein | -2,4 | -5,2 | 2,68E-19 | C |
|  | HORVU6Hr1G049390 | K02704 | psbB | photosystem II CP47 chlorophyll apoprotein | -2,5 | -5,6 | 5,33E-16 | C |
|  | HORVU4Hr1G081790 | K02707 | psbE | photosystem II cytochrome b559 subunit alpha | -6,0 | -63,4 | 5,73E-13 | C |
|  | HORVU5Hr1G065120 | K02707 | psbE | photosystem II cytochrome b559 subunit alpha | -4,9 | -29,1 | 2,94E-35 | C |
|  | HORVU6Hr1G049320 | K02707 | psbE | photosystem II cytochrome b559 subunit alpha | -5,0 | -31,3 | 2,48E-20 | C |
|  | HORVU2Hr1G057700 | K02716 | PsbO | photosystem II oxygen-evolving enhancer protein 1 | -5,4 | -43,4 | 1,87E-31 | N |
|  | HORVU2Hr1G060880 | K02717 | PsbP | photosystem II oxygen-evolving enhancer protein 2 | -5,1 | -35,0 | 1,25E-25 | N |
|  | HORVU4Hr1G000230 | K02717 | PsbP | photosystem II oxygen-evolving enhancer protein 2 | -3,3 | -9,6 | 5,29E-33 | N |
|  | HORVU2Hr1G043960 | K08901 | PsbQ | photosystem II oxygen-evolving enhancer protein 3 | -6,2 | -72,8 | 7,38E-12 | N |
|  | HORVU2Hr1G080260 | K08901 | PsbQ | photosystem II oxygen-evolving enhancer protein 3 | -2,9 | -7,3 | 6,08E-07 | N |
|  | HORVU6Hr1G051650 | K08901 | PsbQ | photosystem II oxygen-evolving enhancer protein 3 | -7,7 | -202,0 | 0,0007 | N |
|  | HORVU1Hr1G017870 | K02703 | psbA | photosystem II P680 reaction center D1 protein [EC:1.10.3.9] | -1,9 | -3,6 | 0,00425 | C |
|  | HORVU2Hr1G043240 | K02703 | psbA | photosystem II P680 reaction center D1 protein [EC:1.10.3.9] | -2,6 | -5,9 | 5,99E-13 | C |
|  | HORVU2Hr1G070570 | K02703 | psbA | photosystem II P680 reaction center D1 protein [EC:1.10.3.9] | -1,5 | -2,8 | 0,000173 | C |
|  | HORVU2Hr1G121820 | K02703 | psbA | photosystem II P680 reaction center D1 protein [EC:1.10.3.9] | -3,5 | -11,5 | 0,000272 | C |
|  | HORVU5Hr1G004630 | K02703 | psbA | photosystem II P680 reaction center D1 protein [EC:1.10.3.9] | -2,5 | -5,8 | 0,000398 | C |
|  | HORVU6Hr1G046820 | K02703 | psbA | photosystem II P680 reaction center D1 protein [EC:1.10.3.9] | -2,3 | -5,1 | 6,88E-07 | C |
|  | HORVU2Hr1G061990 | K02706 | psbD | photosystem II P680 reaction center D2 protein [EC:1.10.3.9] | -2,4 | -5,2 | 2,3E-17 | C |
|  | HORVU2Hr1G121790 | K02706 | psbD | photosystem II P680 reaction center D2 protein [EC:1.10.3.9] | -1,8 | -3,6 | 9,56E-05 | C |
|  | HORVU2Hr1G048390 | K02709 | psbH | photosystem II PsbH protein | -2,6 | -6,0 | 2,93E-08 | N |
|  | HORVU6Hr1G037230 | K02709 | psbH | photosystem II PsbH protein | -2,6 | -6,0 | 2,93E-08 | N |
|  | HORVU6Hr1G049410 | K02709 | psbH | photosystem II PsbH protein | -2,6 | -6,0 | 2,93E-08 | N |
|  | HORVU5Hr1G065130 | K02711 | psbJ | photosystem II PsbJ protein | -5,1 | -34,2 | 4,64E-53 | N |
|  | HORVU6Hr1G049310 | K02711 | psbJ | photosystem II PsbJ protein | -5,1 | -34,2 | 4,64E-53 | N |
|  | HORVU5Hr1G005430 | K02712 | psbK | photosystem II PsbK protein | -4,2 | -18,9 | 0,00223 | N |
|  | HORVU2Hr1G121450 | K02714 | psbM | photosystem II PsbM protein | -2,4 | -5,3 | 3,64E-06 | N |
|  | HORVU6Hr1G049120 | K02714 | psbM | photosystem II PsbM protein | -3,7 | -13,3 | 3,9E-14 | N |
|  | HORVU1Hr1G078140 | K02721 | PsbW | photosystem II PsbW protein | -2,2 | -4,6 | 1,16E-06 | N |
|  | HORVU3Hr1G075870 | K02721 | PsbW | photosystem II PsbW protein | -4,5 | -23,4 | 1,19E-16 | N |
|  | HORVU3Hr1G053780 | K02715 | psbN | PsbN protein | -2,5 | -5,7 | 2,14E-09 | N |
|  | HORVU6Hr1G049400 | K02715 | psbN | PsbN protein | -2,5 | -5,7 | 2,14E-09 | N |
|  | HORVU1Hr1G078380 | K08912 | LHCB1 | light-harvesting complex II chlorophyll a/b binding protein 1 | -11,3 | -2485,2 | 4,14E-07 | N |
|  | HORVU1Hr1G088870 | K08912 | LHCB1 | light-harvesting complex II chlorophyll a/b binding protein 1 | -3,7 | -12,8 | 5,17E-07 | N |
|  | HORVU1Hr1G088900 | K08912 | LHCB1 | light-harvesting complex II chlorophyll a/b binding protein 1 | -11,5 | -2846,1 | 3,54E-05 | N |
|  | HORVU1Hr1G088920 | K08912 | LHCB1 | light-harvesting complex II chlorophyll a/b binding protein 1 | -10,8 | -1768,2 | 3,14E-43 | N |
|  | HORVU1Hr1G089180 | K08912 | LHCB1 | light-harvesting complex II chlorophyll a/b binding protein 1 | -10,4 | -1310,9 | 2,52E-45 | N |
|  | HORVU5Hr1G087250 | K08912 | LHCB1 | light-harvesting complex II chlorophyll a/b binding protein 1 | -3,7 | -13,1 | 1,86E-06 | N |
|  | HORVU5Hr1G109250 | K08912 | LHCB1 | light-harvesting complex II chlorophyll a/b binding protein 1 | -10,5 | -1485,1 | 9,92E-13 | N |
|  | HORVU5Hr1G109260 | K08912 | LHCB1 | light-harvesting complex II chlorophyll a/b binding protein 1 | -10,4 | -1357,6 | 5,1E-37 | N |
|  | HORVU5Hr1G124160 | K08912 | LHCB1 | light-harvesting complex II chlorophyll a/b binding protein 1 | -2,5 | -5,8 | 0,00248 | N |
|  | HORVU6Hr1G016850 | K08912 | LHCB1 | light-harvesting complex II chlorophyll a/b binding protein 1 | -9,5 | -721,8 | 7,49E-35 | N |
|  | HORVU6Hr1G016880 | K08912 | LHCB1 | light-harvesting complex II chlorophyll a/b binding protein 1 | -8,4 | -341,2 | 3,32E-34 | N |
|  | HORVU6Hr1G016890 | K08912 | LHCB1 | light-harvesting complex II chlorophyll a/b binding protein 1 | -7,6 | -193,7 | 1,84E-48 | N |
|  | HORVU6Hr1G091650 | K08912 | LHCB1 | light-harvesting complex II chlorophyll a/b binding protein 1 | -11,9 | -3743,9 | 5,74E-07 | N |
|  | HORVU5Hr1G082420 | K08913 | LHCB2 | light-harvesting complex II chlorophyll a/b binding protein 2 | -6,2 | -74,1 | 1,83E-23 | N |
|  | HORVU2Hr1G040780 | K08914 | LHCB3 | light-harvesting complex II chlorophyll a/b binding protein 3 | -6,1 | -68,9 | 1,21E-20 | N |
|  | HORVU2Hr1G079920 | K08917 | LHCB6 | light-harvesting complex II chlorophyll a/b binding protein 6 | -5,7 | -51,8 | 1,24E-24 | N |
|  | HORVU5Hr1G054000 | K14172 | LHCB7 | light-harvesting complex II chlorophyll a/b binding protein 7 | -1,9 | -3,8 | 0,000828 | N |

N – nuclear, C – chloroplast genome
