# Supplementary material for: Genetic and Physiological Dissection of Photosynthesis in Barley Exposed to Drought Stress
Source: Int J Mol Sci. 2019 Dec 16;20(24):6341. doi: 10.3390/ijms20246341 (PMC6940956; doi:10.3390/ijms20246341)
Supplement: Supplementary file 1 [file ijms-20-06341-s001.zip › ADG_supplementary materials legend.docx]

Supplementary Data Legend

Genetic and physiological dissection of photosynthesis in barley exposed to drought stress

Agata Daszkowska-Golec^1^*, Anna Collin^1^, Krzysztof Sitko^1^, Agnieszka Janiak^1^, Hazem M. Kalaji^2^ and Iwona Szarejko^1^

^1^University of Silesia in Katowice, Faculty of Natural Sciences, Institute of Biology, Biotechnology and Environmental Protection, Jagiellońska 28, 40-032 Katowice, Poland; [agata.daszkowska@us.edu.pl](mailto:agata.daszkowska@us.edu.pl); [anna.skubacz@us.edu.pl](mailto:anna.skubacz@us.edu.pl); [agnieszka.janiak@us.edu.pl](mailto:agnieszka.janiak@us.edu.pl); [iwona.szarejko@us.edu.pl](mailto:iwona.szarejko@us.edu.pl); [krzysztof.sitko@us.edu.pl](mailto:krzysztof.sitko@us.edu.pl)

^2^ Warsaw University of Life Sciences (WULS-SGGW), Institute of Biology, Department of Plant Physiology, Nowoursynowska 159, 02-776Warszawa, Poland, [hazem@kalaji.pl](mailto:hazem@kalaji.pl)

***** Correspondence: [agata.daszkowska@us.edu.pl](mailto:agata.daszkowska@us.edu.pl); Tel.: +48 32 2009 360

**Supplementary materials legend:**


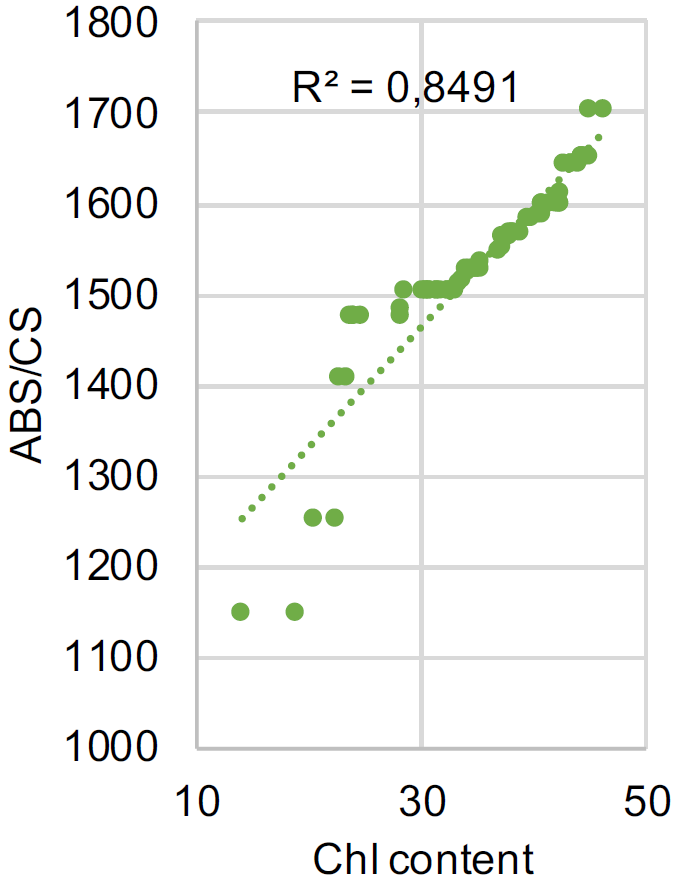


**Figure S1**. Positive correlation between ABS/CS (Absorption energy flux per illuminated cross-section) and chlorophyll content (Chl content).

**Table S1**. Significantly enriched gene ontology (GO) terms of down- and up-regulated genes identified using RNaseq approach (FDR≤0.05); BP – biological process, CC – cellular component, MF – molecular function.

**Table S2.** DEGs identified using RNA-seq as photosynthesis related downregulated under drought in barley

**Table S3.** Statistical significance for putative regulatory pairs between DEGs encoding transcription factors and predicted targets identified within photosynthesis-related genes.

**Table S4**. The analysis of photosynthesis related parameters measured with the use of JIP-test in three barley cultivars exposed to drought stress. Different letters correspond to statistical significance between genotypes and conditions studied. Statistical analyses were performed using ANOVA (P<0.05) followed by Tukey’s honestly significant difference test (Tukey HSD test) (P<0.01).

**Table S5**. The analysis of photosynthesis related parameters measured with the use of JIP-test in three barley cultivars treated with exogenously applied ABA. Different letters correspond to statistical significance between genotypes and conditions. Statistical analyses were performed using 2-way ANOVA (P<0.05) followed by Tukey’s honestly significant difference test (Tukey HSD test) (P<0.01).

**Table S6**. The correlation co-efficient between physiological parameters derived from JIP-test after drought exposure.
