# Supplementary figures and images for: Genetic and Physiological Dissection of Photosynthesis in Barley Exposed to Drought Stress
Source: Int J Mol Sci. 2019 Dec 16;20(24):6341. doi: 10.3390/ijms20246341 (PMC6940956; doi:10.3390/ijms20246341)

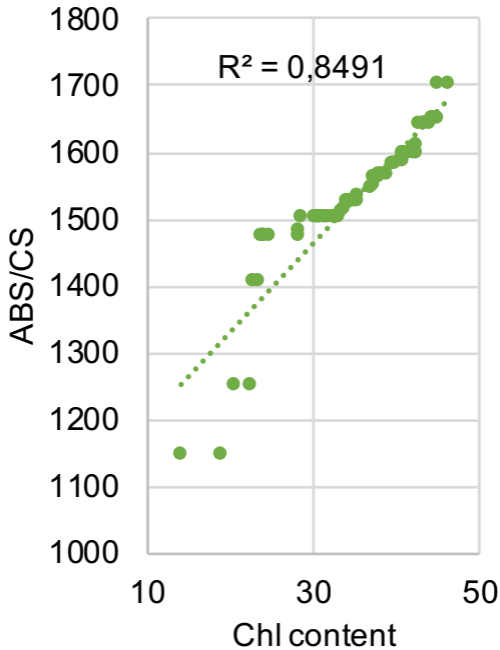

Supplement: Supplementary file 1 [file ijms-20-06341-s001.zip › ADG_et al_Figure-S1.pdf]
